# The relationship between intraflagellar transport and upstream protein trafficking pathways and macrocyclic lactone resistance in *Caenorhabditis elegans*

**DOI:** 10.1093/g3journal/jkae009

**Published:** 2024-01-16

**Authors:** Robert A Brinzer, Alan D Winter, Antony P Page

**Affiliations:** School of Biodiversity, One Health and Veterinary Medicine, University of Glasgow, Scotland G61 1QH, UK; School of Biodiversity, One Health and Veterinary Medicine, University of Glasgow, Scotland G61 1QH, UK; School of Biodiversity, One Health and Veterinary Medicine, University of Glasgow, Scotland G61 1QH, UK

**Keywords:** *Caenorhabditis elegans*, macrocyclic lactone, ivermectin, moxidectin, anthelmintic resistance, xenobiotic resistance, amphids, ciliogenesis, intraflagellar transport

## Abstract

Parasitic nematodes are globally important and place a heavy disease burden on infected humans, crops, and livestock, while commonly administered anthelmintics used for treatment are being rendered ineffective by increasing levels of resistance. It has recently been shown in the model nematode *Caenorhabditis elegans* that the sensory cilia of the amphid neurons play an important role in resistance toward macrocyclic lactones such as ivermectin (an avermectin) and moxidectin (a milbemycin) either through reduced uptake or intertissue signaling pathways. This study interrogated the extent to which ciliary defects relate to macrocyclic lactone resistance and dye-filling defects using a combination of forward genetics and targeted resistance screening approaches and confirmed the importance of intraflagellar transport in this process. This approach also identified the protein trafficking pathways used by the downstream effectors and the components of the ciliary basal body that are required for effector entry into these nonmotile structures. In total, 24 novel *C. elegans* anthelmintic survival-associated genes were identified in this study. When combined with previously known resistance genes, there are now 46 resistance-associated genes that are directly involved in amphid, cilia, and intraflagellar transport function.

## Introduction

Parasitic nematodes place a highly significant and heavy disease burden on infected plants and animals causing annual global yield and productivity losses in excess of $100 billion (Grisi *et al*. 2014; Singh *et al*. 2015) and, in addition, requiring over $20 billion annually to treat with anthelmintics (Abongwa *et al*. 2017). Currently available broad-spectrum anthelmintics are from a limited range of chemical families (Abongwa *et al*. 2017), and resistance to one or more classes is becoming widespread in field populations (Crook *et al*. 2016) jeopardizing food security and human health. Therefore, until new anthelmintic classes are developed, it is necessary to prolong the efficacy of existing drugs by finding ways to suppress resistance.

The macrocyclic lactones such as ivermectin (an avermectin) and moxidectin (a milbemycin) are the most commonly administered anthelmintics due to their low cost and high persistent efficacy (McArthur and Reinemeyer 2014); however, the rapid spread of resistance is beginning to render them ineffective (Crook *et al*. 2016). There has been an observed correlation between macrocyclic lactone resistance caused by reduced uptake and defects in amphid morphology in *Caenorhabditis elegans* with several causative genes being associated with dye-filling, chemosensation, osmosensation, dauer formation, and mechanosensation defective phenotypes (Dent *et al*. 2000; Urdaneta-Marquez *et al*. 2014; Page 2018). Amphid morphology and dye-filling defects have also been noted in field populations of *Haemonchus contortus* that are resistant to macrocyclic lactones (Freeman *et al*. 2003; Urdaneta-Marquez *et al*. 2014). Recently, it has been found that some amphid morphology-associated genes may also influence intestinal anthelmintic exporting P-glycoprotein expression in an NHR-8-dependent manner (Guerrero *et al*. 2021).

The amphid sensilla consist of 2 pairs of 12–13 neurons (12 in *C. elegans*), which have nonmotile cilia enriched in G protein-coupled receptors on the dendrites that are exposed to the environment through pores in the cuticle (Perkins *et al*. 1986; Brear *et al*. 2014; Vidal *et al*. 2018; Hong *et al*. 2019), and function as the primary sensory organ for environmental stimuli (chemical, ion and osmotic gradients, temperature, pheromones, and noxious compounds). Sensory inputs are processed by the nerve ring leading to output motor neuron-mediated responses (Schafer 2016; Cook *et al*. 2019; Hong *et al*. 2019) in a 200–300 neuron nervous system whose layout is highly conserved between nematode species. Ciliogenesis of sensory cilia utilizes assembly pathways that are conserved throughout Eukaryota where a centriole-derived basal body anchors to the cell membrane restricting the local diffusion of proteins and lipids and organizes microtubules (Sung and Leroux 2013). These microtubules are then used for the delivery of lipids and proteins to the growing cilia by intraflagellar transport (IFT) complexes that travel along the microtubules using dynein and kinesin motors (Rosenbaum and Witman 2002; Sung and Leroux 2013; Prevo *et al*. 2017).

Macrocyclic lactones function by paralyzing the central nervous system, which eventually leads to death, through interaction with multiple subunits of the glutamate-gated chloride channel primary target, as well as multiple secondary targets, thereby resulting in constitutive activation (Chen and Kubo 2018). As nematodes have limited capacity for phase I detoxification (functionalization and oxidation) of macrocyclic lactones (Vokřál *et al*. 2013; Yilmaz *et al*. 2019), resistance relies on increased phase II conjugation and efflux (James and Davey 2009), target site insensitivity, or reduced drug uptake (Dent *et al*. 2000). However, all identified and candidate resistance genes that interact directly with macrocyclic lactones or their metabolites function downstream of macrocyclic lactone uptake (Dent *et al*. 2000; James and Davey 2009; Ménez *et al*. 2016). The macrocyclic lactones lack the chemical properties that would allow them to spontaneously cross biological membranes (Escher *et al*. 2008) meaning that uptake is dependent on the ability of the biological systems of the organism to accumulate appropriate concentrations in the target tissues; however, the mechanism and associated genes involved in uptake are still unknown or poorly defined.

This current study uses a mechanistic approach to investigate cellular processes associated with previously discovered resistance genes, in combination with targeted resistance screens in *C. elegans*, to identify the roles played by anterograde and retrograde IFT in the ciliary distal segment of the amphid neurons in the resistance to macrocyclic lactones (ivermectin and moxidectin). Pathways involved in trafficking ciliary proteins to and from the ciliary gate of the basal body were also investigated, revealing that the UNC-101- and UNC-119-mediated secretion pathways and the polarizers of axon–dendrite protein sorting UNC-33 and UNC-44 are important components involved in macrocyclic lactone resistance, whereas the RAB-35 recycling pathway plays a downstream role. A whole-genome sequencing approach was applied to map candidates from a forward genetic screen for resistance to macrocyclic lactones, and in combination with a targeted resistance screen, 24 novel anthelmintic survival-associated genes were uncovered in *C. elegans*.

## Methods

### Chemicals

Suppliers and catalog numbers of all reagents used are listed in the Supplementary Methods.

### Nematode strains

Putative orthologs of key basal body genes for which there was no primary literature were chosen using a combination of Protein BLAST (https://blast.ncbi.nlm.nih.gov/Blast.cgi) and the MARRVEL (Wang *et al*. 2017) and AceView (Thierry-Mieg and Thierry-Mieg 2006) databases.

TM prefixed strains were obtained from the National BioResource Project, Japan while all other strains used were purchased from the *C. elegans* Genetics Centre, USA. All strains were maintained on *Escherichia coli* OP50-1-inoculated Nematode Growth Medium (NGM) plates following standard protocols (http://www.wormbook.org/toc_wormmethods.html). Strains used in this study are listed in the Supplementary Methods.

### Anthelmintic resistance assays

Anthelmintic stock solutions were prepared as follows: 10 µM ivermectin stock was made by the serial dilution of a 10 mM stock using DMSO as a solvent for both stocks; 10 µM moxidectin stock was prepared using the same procedure as ivermectin. Stock solutions were dispensed into 1-ml aliquots and stored at −20°C.

NGM plates containing anthelmintics were produced by adding volumes of anthelmintic stock solution to cooled molten NGM agar (50°C) before mixing and pouring onto 3-cm petri dishes. The volume of the anthelmintic stock solution added never exceeded 0.3% of the final volume. Anthelmintic plate concentrations used were 10 nM ivermectin and 5 and 10 nM moxidectin based on resistance threshold criteria used for gastrointestinal nematodes (Kaplan *et al*. 2007; Crook *et al*. 2016). Plates were inoculated with 50-µl OP50-1 24 h before starting assays.

To determine ivermectin and moxidectin resistance, survival assays were performed by picking 5 L4 worms of the strain to be tested onto each plate with 2 biological and 2 technical replicates. Growth and mortality were inspected every 48 h using a light microscope. A strain was considered resistant (+) if the F1 generation reached adulthood compared to susceptible strains (−), which showed paralysis and growth arrest and F1s failed to reach adulthood. The wild-type N2 strain was used as a susceptible negative control and DA1316 (*ad1305*; *vu227*; *pk54*) was used as a resistant positive control.

### DiI dye-filling assays and microscopy

Worms were washed from populated plates using M9 buffer (3-g KH_2_PO_4_, 6-g Na_2_HPO_4_, 5-g NaCl, and 1 mM MgSO_4_/L) and collected in 1.5-ml eppendorfs. Samples were pelleted by centrifugation at 7,000 rpm for 10 s to allow the removal of the supernatant. Two washes with M9 were performed before applying 10-µg/ml DiI (1,1ʹ-dioctadecyl-3,3,3ʹ,3ʹ-tetramethylindocarbocyanine perchlorate) dye in M9 buffer for 30 min. Samples were then washed twice with M9 before incubating at 21°C for 2 h to allow worms to clear their gut of bacteria and dislodge DiI adhered to the cuticle before performing 2 more washes in M9. Worms were pelleted and supernatant removed before transfer to an empty petri dish using a pipette and then picking 20–30 specimens onto prepared microscope slides. Slides were coated with a pad of 2% agar with 1% sodium azide and wet with 10 µl of M9 containing 0.2% sodium azide, and then coverslips were sealed with a thin layer of petroleum jelly.

**Fig. 1.**
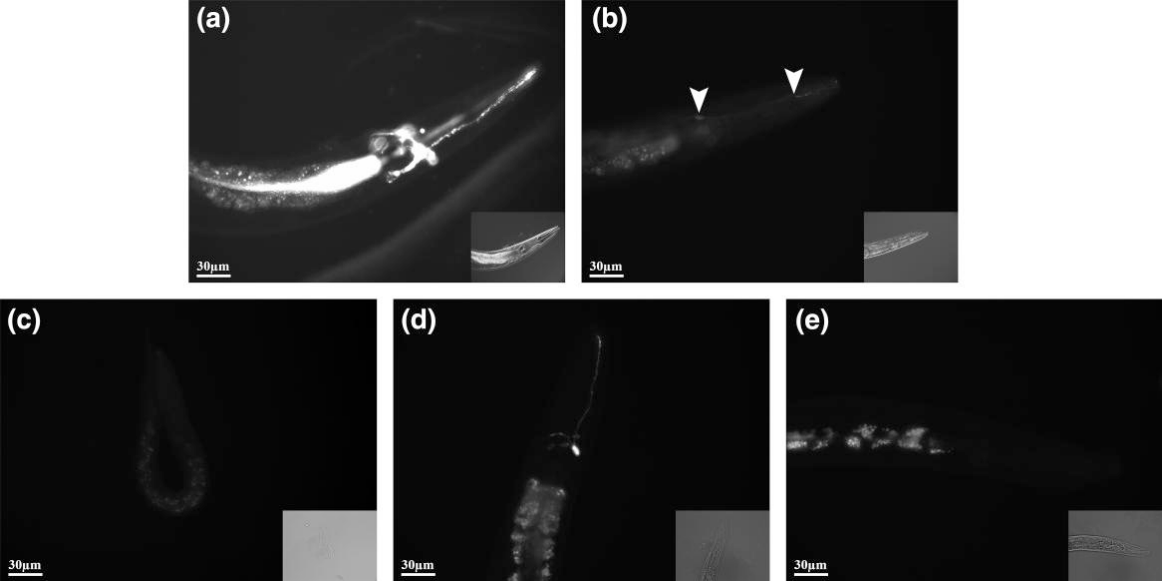
Representative images of DiI phenotypes at 250× magnification. DiI, 1,1ʹ-dioctadecyl-3,3,3ʹ,3ʹ-tetramethylindocarbocyanine perchlorate. a) N2: DiI dye-filling positive, b) *ifta-1*(*nx61*): weak DiI dye-filling positive, c) *dyf-2*(*m160*): DiI dye-filling negative and *c14h10.2*(*tm10737*): novel Dyf mutant that has variable DiI dye filling with d) weak positive individuals in a predominantly e) negative population. Individuals were photographed using a DIC filter (lower right inset image) to highlight the position and orientation of the worm and a FITC filter (main image) to visualize fluorescence. Areas of fluorescence for weak phenotypes are highlighted with arrows.

Slides were viewed using a Zeiss Axioskop 2 Plus microscope fitted with a Zeiss Mercury HBO 100 Lamphouse and Zeiss AxioCam camera with images taken using the accompanying AxioVision software. All images were taken at 250× magnification. Control images of worms were taken using a differential interference contrast (DIC) filter, 0.5-s exposure time, and the minimum setting for the internal light source while DiI staining was viewed and imaged using a fluorescein isothiocyanate (FITC) filter, 1-s exposure time, and illumination by the mercury lamp. A minimum of 10 individuals of each strain were observed under FITC conditions to score the average intensity of DiI staining (negative or abnormal dye filling [−] or positive [+]). Representative DIC and FITC images for DiI staining patterns in each category are shown in Fig. 1 while images for individual strains are available upon request.

### EMS mutagenesis and whole-genome sequencing

*C. elegans* L4 stage N2 strain worms were exposed to 50 mM ethyl methanesulfonate (EMS) for 4 h at 20°C following standard mutagenesis procedures (Brenner 1974) and then allowed to recover on OP50-1-seeded NGM plates overnight. Worms were then handled according to Page (2018) selecting for 10 nM moxidectin resistance (see Supplementary Methods for details). Lines were then characterized for DiI dye-filling and ivermectin, albendazole, and levamisole cross-resistance.

From the 14 resulting moxidectin-resistant lines, 5 were selected, and together with uncharacterized ivermectin-resistant lines, TP236(*ka30*), TP241(*ka35*), TP272(*ka64*), and TP274(*ka66*) from a previous study (Page 2018) were processed for single-nucleotide polymorphism (SNP) mapping. SNP mapping was carried out as described in Doitsidou *et al*. (2010) using MiModD tools on the public instance of the Galaxy platform (https://usegalaxy.org) (Jalili *et al*. 2020) (see Supplementary Methods for details). Genomic DNA was extracted using a Gentra Puregene Core Kit A (Qiagen, UK) kit before cleanup and concentration using a Genomic DNA Clean & Concentrator-25 (Zymo Research, USA) kit. Samples were sent for whole-genome sequencing to the Glasgow Polyomics facility, University of Glasgow where libraries were prepared with a TruSeq Nano DNA LT Sample Prep Kit (Illumina), quality controlled on a 2100 Bioanalyzer (Agilent), and run on an Illumina MiSeq platform using 300-bp paired-end reads.

## Results

Outcomes of dye-filling and macrocyclic lactone survival assays are listed in Table 1. Strains tested that did not show a phenotype of interest are included in Supplementary Table 1. The relationship between resistance, IFT, protein trafficking, cilia, and dye filling is summarized below.

**Table 1.** Many *C. elegans* mutants for ciliary proteins are resistant to ivermectin and moxidectin.

Gene (homology)	Strain (allele)	DYF	IVM R	MOX R
**Transcription factor mutants (2/4)**				
*daf-19* (RFX transcription factor)	DR86(*m86*)	−	+	+
*hlh-4* (achaete-scute transcription factor)	TM604(*tm604*)	−	+	+
**Cell migration/adhesion defect mutants (2/6)**				
*dyf-7* (ZP protein)	SP1735(*m537*)	−	+	+
*mec-8* (RRM domain/splice factor)	CB398(*e398*)	+	+	+
**Amphid channel morphology mutants (1/2)**				
*daf-6* (PTCHD-1/4 ortholog)	CB1377(*e1377*)	−	+	+
**Protein secretion/trafficking defect mutants (9/52)**				
*arl-13* (ARL13B ortholog)	TM1745(*tm1745*)	+	−	+
*dyf-5* (map kinase)	SP1745(*mn400*)	−	+	+
*dyf-18* (CDK-8/19/20 ortholog)	ET100(*ok200*)	−	−	+
*osta-1* (SLC51A ortholog)	TM5255(*tm5255*)	+	−	+
*rab-35* (RAB family)	RT206(*b1013*)	+	+	+
*unc-33* (CRMP1 ortholog, filamin binding)	CB1193(*e1193*)	+	+	+
*unc-44* (ANK2/ANK3 ortholog)	CB1197(*e1197*)	−	+	+
*unc-101* (AP1M1 ortholog)	PS529(*sy108*)	−	+	+
*unc-119* (HRG4 ortholog)	CB4845(*e2498*)	−	+	+
**Ciliary nucleation and region identity mutants (8/44)**				
*c14h10.2* (JAKMIP3 ortholog, putative CEP123 ortholog)	TM10737(*tm10737*)	−	+	+
*che-10* (rootelin, IFT)	CB3329(*e1809*)	−	+	+
*che-12* (TOGARAM1 ortholog, IFT)	CB3332(*e1812*)	−	+	+
*dyf-17* (MAGEL2 like)	EG175(*ox175*)	−	+	+
*dyf-19* (FBF1 ortholog)	ZP541(*jhu455*)	−	+	+
*hyls-1* (hydrolethalus syndrome ortholog)	TM3067(*tm3067*)	−	+	+
*nphp-4* (nephrocystin 4 ortholog)	TM925(*tm925*)	−	+	+
*yap-1* (WWTR1 ortholog, has CEP164 homology)	TM1416(*tm1416*)	+	−	+
**Microtubule mutants (1/2)**				
*dyf-10* (α-tubulin homolog)	SP1709(*e1383*)	−	+	+
**Dynein and kinesin motor mutants (4/22)**				
*che-3* (Dynein HC avr-1, IFT)	CB1124(*e1124*)	−	+	+
*dhc-3* (Dynein HC, IFT)	TP239(*ka33*)	−	+	+
*osm-3* (kinesin family, IFT)	PR802(*p802*)	−	+	+
*xbx-1* (DYNC2LI1 ortholog)	JT11069(*ok279*)	−	+	+
**IFT-A complex mutants (5/6)**				
*che-11* (IFT140 homolog, IFT)	CB3330(*e1810*)	−	+	+
*daf-10* (IFT122A homolog, WD repeat, IFT)	CB1387(*e1387*)	−	+	+
*dyf-2* (IFT144 homolog, WRD19, IFT)	SP1234(*m160*)	−	+	+
*ift-43* (IFT43 homolog, IFT)	TM8137(*tm8137*)	+	−	+
*ifta-1* (IFT122B homolog, WDR35, IFT)	MX124(*nx61*)	−	+	+
**IFT-B complex mutants (13/16)**				
*che-2* (IFT80 homolog, G-protein, WD repeat)	CB1033(*e1033*)	−	+	+
*che-13* (IFT57/Hippi)	CB3323(*e1815*)	−	+	+
*dyf-1* (IFT70 homolog, IFT)	SP1205(*mn335*)	−	+	+
*dyf-3* (IFT38 homolog, CLUAP protein, IFT)	SP1603(*m185*)	−	+	+
*dyf-6* (IFT46 homolog, IFT)	SP1712(*m175*)	−	+	+
*dyf-11* (IFT54 homolog, IFT)	SP1713(*mn392*)	−	+	+
*dyf-13* (IFT56 homolog, IFT)	SP1678(*mn396*)	+	+	+
*ift-20* (IFT20 homolog, IFT)	RB2353(*ok3191*)	−	+	+
*ift-74* (IFT72/74 homolog, IFT)	VC2140(*ok2866*)	+	+	+
*osm-1* (IFT172 homolog, WD repeat, IFT)	PR808(*p808*)	−	+	+
*osm-1* (IFT172 homolog, WD repeat, IFT)	PR816(*p816*)	−	+	+
*osm-5* (IFT88 homolog, polaris, IFT)	PR813(*p813*)	−	+	+
*osm-6* (IFT52 homolog, IFT)	PR811(*p811*)	−	+	+
*rab-28* (RAB family, IFT27 homolog)	RB2484(*ok3424*)	−	+	+
**Bardet–Biedl syndrome complex mutants (6/10)**				
*bbs-1* (BBS1 ortholog, IFT)	VC837(*ok1111*)	−	+	+
*bbs-2* (BBS2 ortholog, IFT)	VC1569(*ok2053*)	−	+	+
*bbs-8* (BBS8 ortholog, TPR protein, IFT)	MX52(*nx77*)	−	+	+
*bbs-9* (BBS9 ortholog, IFT)	VC1062(*gk471*)	−	+	+
*k07c11.10* (BBS10 ortholog)	TM3304(*tm3304*)	+	+	+
*osm-12* (bbs7, IFT)	MT3645(*n1606*)	−	+	+
**IFT cargo mutants (1/14)**				
*osm-9* (TRPV5/6 family)	CX10(*ky10*)	+	+	+
**OSM-9 interacting/associated proteins (1/12)**				
*npr-1* (NPY1R ortholog)	CX4148(*ky13*)	+	−	+
**Other sensory mutants (3/10)**				
*inx-19* (innexin homolog)	CX6161(*ky634*)	−	+	+
*unc-7* (innexin homolog)	CB5(*e5*)	+	+	+
*unc-9* (innexin homolog)	CB101(*e101*)	+	+	+

Numbers of positive hits under each category out of the total genes tested are in parentheses.

Underline: previously identified mutants that are resistant to one of the anthelmintics tested.

Dyf, DiI amphid dye filling; IVM R, ivermectin resistance; MOX R, moxidectin resistance; +, dye filling identical to the wild type/resistant (includes weak resistance); −, dye filling defective (includes weak dye filling)/susceptible; IFT, intraflagellar transport component homology.

### IFT complex subunits

Of the previously 34 identified ivermectin resistance genes (Dent *et al*. 2000; Urdaneta-Marquez *et al*. 2014; Page 2018), 16 encode for proteins of the IFT-A complex, IFT-B complex, and the BBSome, all of which are interacting multiprotein complexes involved in IFT (Rosenbaum and Witman 2002; Prevo *et al*. 2017). Orthologs of the remaining 14 known, but untested, subunits of these complexes and an ortholog of the chaperone protein BBS10, were investigated for anthelmintic resistance. Out of the 15 genes tested, mutant alleles for 8 showed resistance to ivermectin (Table 1). Within the IFT-A complex mutants, the IFTA-1 dynein-interacting protein mutant was found to be strongly resistant to ivermectin (Table 1), while mutants for the dynein-loading proteins IFT-43 and IFT-139 remained susceptible (Supplementary Table 1). From the IFT-B complex mutants, the Golgi vesicle sorting protein IFT-20 and the tubulin delivery protein IFT-74 mutants were only weakly resistant to ivermectin whereas the IFT27 ortholog RAB-28 mutant was highly resistant (Table 1). Mutants for the core BBSome proteins BBS-2 and BBS-9 and the BBS10 ortholog K07C11.10 all displayed strong ivermectin resistance (Table 1) while those for the cargo adapter subunits BBS-4 and BBS-5 were susceptible (Supplementary Table 1).

### Known IFT cargoes

As the primary function of IFT is the delivery of ciliary proteins, genes for known IFT cargo proteins were tested for ivermectin resistance to identify downstream effectors of resistance. Of the 14 cargo protein-encoding genes tested, only the CX10(*ky10*) mutant of *osm-9* was found to exhibit resistance (Table 1); however, this finding was not replicated with the VC1262(*ok1677*) and JY190(*yz6*) *osm-9* mutant strains (Supplementary Table 1) indicating that perhaps resistance is caused by an unrelated, uncharacterized, mutation in the CX10(*ky10*) strain. The ciliary membrane protein cargo adaptor *tub-1*(*ok1972*) mutant was found to be susceptible to ivermectin (Supplementary Table 1), supporting the hypothesis that the downstream effector for ivermectin resistance must be delivered by another secretion pathway.

### Protein trafficking pathways

To gain insight into the trafficking of the downstream effectors for ivermectin resistance, known ciliary protein secretion pathways upstream of the IFT and ciliary membrane protein removal pathways were investigated. The clathrin adapter protein-1 ortholog involved in Golgi vesicle secretion UNC-101, the CRMP1 ortholog involved in polarizing axon–dendrite sorting UNC-33, the ANK2/ANK3 ortholog involved in polarizing axon–dendrite sorting UNC-44, UNC-119 that inserts myristoylated proteins into the cell membrane, and RAB-35 that regulates early endosome recycling were all involved in causing ivermectin resistance when mutated (Table 1). Mutants for the 2 SNAP25 family protein-encoding genes *aex-4* and *ric-4* were found to be susceptible (Supplementary Table 1), supporting the contention that the downstream effector, which causes ivermectin resistance when absent, must be delivered via vesicle fusion using the essential SNAP-29 protein. All the genes so far tested that are involved in endocytosis, designation to lysosomal degradation, early endosome maturation, extracellular vesicle formation, synaptic vesicle fusion, and other post-Golgi transport complexes did not confer ivermectin resistance (Supplementary Table 1). Intriguingly, mutants for the RAB-8 and RAB-10 exocytosis regulators, which have roles in crossing the ciliary gate, were likewise susceptible to this drug (Supplementary Table 1).

Dyneins and kinesins play an important role in protein trafficking and IFT with *osm-3*(*p802*), *che-3*(*e1124*), and *dhc-3*(*ka33*) already being associated with ivermectin resistance (Dent *et al*. 2000; Page 2018); therefore, additional members of these families were investigated. Of the 20 genes tested, only mutations in the dynein light-intermediate chain *xbx-1* resulted in ivermectin resistance (Table 1). Mutant alleles for all 3 genes encoding the IFT heterotrimeric kinesin (*kap-1*, *klp-11*, and *klp-20*) and the axonal kinesin *unc-104* had no impact on ivermectin resistance (Supplementary Table 1).

### The ciliary gate

The ciliary gate of the basal body acts as a physical barrier at the base of the cilia that selectively allows the passage of ciliary proteins. Components of the ciliary gate (some putative) were therefore investigated to uncover those required to deliver downstream effectors associated with ivermectin resistance. The MAGEL2-like protein DYF-17, the distal appendage-interacting subunit of the basal body HYLS-1, the FBF1 ortholog DYF-19, the transition fiber subunit NPHP-4, and the JAKMIP3 ortholog with CEP123 homology C14H10.2 were all found to be involved in maintaining ivermectin susceptibility (Table 1) although some *nphp-4*(*tm925*) individuals showed incomplete penetrance of the resistance phenotype. Mutants for all other transition fiber genes, putative subdistal appendage proteins, putative ESCRT complex, exocyst vesicle, TRAPP complex, and Rab family-interacting basal body subunits and orthologs of the ARMC9/TOGARAM1 complex were all tested and found to have no impact on ivermectin resistance (Supplementary Table 1).

### Cell migration, amphid formation, ciliogenesis, and ciliated neuron-enriched genes tested

As gross morphological defects to amphid neurons, their cilia, and the amphid channel invariably cause ivermectin resistance, some transcription factors that determine amphid neuron cell fate and the proteins involved in axon guidance and lumen formation were assessed for a role in ivermectin resistance. Of the 5 genes tested, only mutant alleles for the ADL neuron determining transcription factor *hlh-4* and the lumen endocytosis regulator *daf-6* were found to cause resistance to ivermectin (Table 1).

Some genes involved in gap junction formation (*unc-7* and *unc-9*), mechanosensation (*mec-1* and *mec-8*), and osmotic avoidance (*osm-1*, *osm-3*, *osm-5*, *osm-6*, and *osm-12*) have been reported to cause ivermectin resistance (Dent *et al*. 2000; Page 2018), so additional genes in those phenotype categories along with several cilium-enriched membrane proteins (Blacque *et al*. 2005; Kunitomo *et al*. 2005) were likewise investigated. Of the genes from this grouping that have been tested, only the gap junction innexin *inx-19(ky634)* mutant displayed resistance to both ivermectin and moxidectin (Table 1) while no resistance was observed in both *mec-1* strains tested (Supplementary Table 1).

### Amphidal dye-filling defect correlation with ivermectin resistance

It has previously been found that there is a correlation between ivermectin resistance and dye-filling defects (Page 2018), so the full extent of this relationship was examined. Of previously known ivermectin resistance genes, the mutant alleles *daf-19*(*m86*), *dyf-7*(*m537*), *che-12*(*e1812*), *dyf-10*(*e1383*), *che-3*(*e1124*), *dhc-3*(*ka33*), *osm-3*(*p802*), *che-11*(*e1810*), *daf-10*(*e1387*), *dyf-2*(*m160*), *che-2*(*e1033*), *che-13*(*e1815*), *dyf-1*(*mn335*), *dyf-3*(*m185*), *dyf-6*(*m175*), *dyf-7*(*m537*), *dyf-10*(*e1383*), *dyf-11*(*mn392*), *osm-1*(*p808*), *osm-1*(*p816*), *osm-3*(*p802*), *osm-5*(*p813*), and *osm-6*(*p811*) were dye-filling negative; *bbs-8*(*nx77*), *che-10*(*e1809*), *bbs-1*(*ok1111*), *osm-12*(*n1606*), and *dyf-5*(*mn400*) exhibited weak dye filling (Table 1); *che-1*(*p672*), *che-1*(*ot75*), *che-6*(*e1126*), *dyf-13*(*mn396*), *mec-1*(*e1066*), *mec-8*(*e398*), *unc-7*(*e5*), and *unc-9*(*e101*) were dye-filling positive (Supplementary Table 1); and *che-14*(*e1960*) exhibited highly variable degrees of dye filling between individuals. Among the novel ivermectin resistance genes identified in the present study, the mutant alleles *unc-101*(*sy108*), *daf-6*(*e1377*), *ift-20*(*ok3191*), *rab-28*(*ok3424*), *bbs-2*(*ok2053*), *dyf-19*(*jhu455*), and *inx-19*(*ky634*) were all dye-filling negative; *hlh-4*(*tm604*), *unc-119*(*e2498*), *hyls-1*(*tm3067*), *nphp-4*(*tm925*), *ifta-1*(*nx61*), and *bbs-9*(*gk471*) displayed weak dye filling (Table 1); *rab-35*(*b1013*), *unc-33*(*e1193*), *ift-74*(*ok2866*), and *k07c11.10*(*tm3304*) were dye-filling positive (Table 1); and *c14h10.2*(*tm10737*), *dyf-17*(*ox175*), *unc-44*(*e1197*), and *xbx-1*(*ok279*) had highly variable degrees of dye filling between individuals, with *c14h10.2*(*tm10737*), *dyf-17*(*ox175*), and *unc-44*(*e1197*) being predominantly dye-filling negative (Table 1). The *tag-278*(*gk382*) mutant also showed highly variable degrees of dye filling between individuals but showed no resistance to any of the tested anthelmintics (Supplementary Table 1).

Processes that are essential for ciliogenesis and cilium maintenance showed a strong correlation between the extent of dye-filling defects and the strength of ivermectin resistance although *mec-8*(*e398*), *hyls-1*(*tm3067*), *ifta-1*(*nx61*), and *bbs-9*(*gk471*) defied the trend by showing strong resistance despite having weak dye filling. Mutants for proteins that are involved in trafficking ciliary membrane proteins along the axon such as UNC-33 and proteins that function downstream of IFT, including RAB-35 and helper/regulatory proteins like K07C11.10, showed no correlation. This indicates that although DiI dye filling and ivermectin susceptibility require effector delivery to the cilia through shared pathways, both processes do not necessarily use the same effector.

### Observed cross-resistances to moxidectin

Candidate genes were also tested for moxidectin (a milbemycin) resistance to examine possible cross-resistance. Mutants for all genes that were ivermectin resistant were also resistant to moxidectin, indicating as expected, shared mechanisms and also similar levels of resistance to the 2 drugs. Mutants for the kinase DYF-18 that plays a role in ciliogenesis and IFT, a regulator of ciliary protein trafficking OSTA-1, the small GTPase nucleotide exchange factor involved in ciliogenesis ARL-13, a WWTR1 ortholog with CEP164 homology YAP-1, and the IFT-A complex dynein-loading protein IFT-43, however, showed moxidectin resistance but not ivermectin resistance (Table 1).

### Whole-genome sequencing of mutants from forward genetic screens

**Fig. 2.**
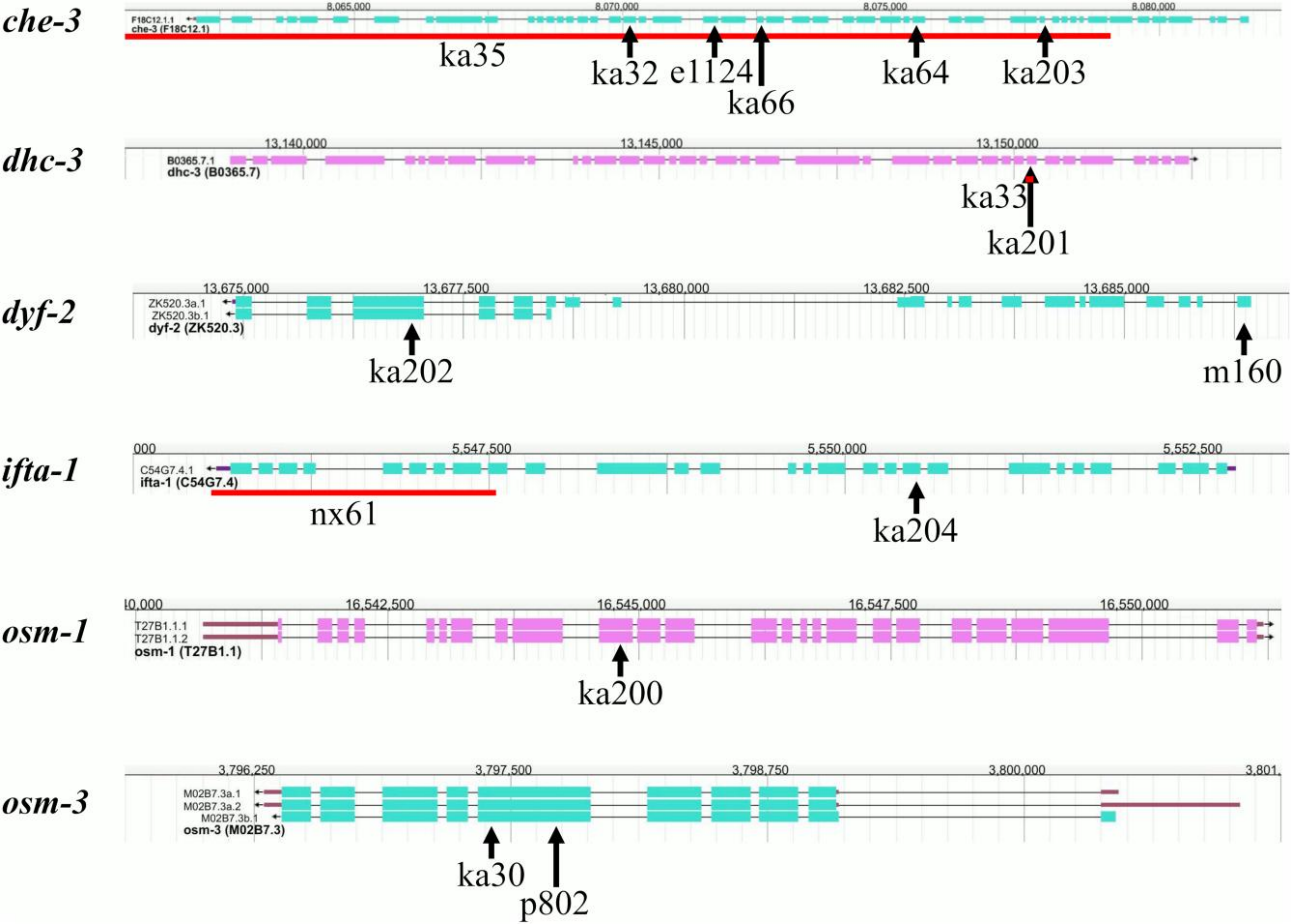
Position of novel and tested alleles in resistance genes identified by whole-genome sequencing. Transcript structures and positions of genes were obtained from WormBase (https://wormbase.org) (JBrowse version: WS281; genome build WBcel235). Arrows above alleles point to their location in the genomic sequence. Solid lines directly above alleles span the length of deletions. Alleles featured (*name* = chr-number: position nt-change [aa-change]) are *e1124* = I: 8,071,718 G > A (Q > Stop); *ka30* = IV: 3,797,404 G > A (Q > Stop); *ka32* = I: 8,070,133 C > T (G > R); *ka33* = V: 13,150,172–13,150,276 deletion; *ka35* = I: 8,058,869–8,079,083 deletion; *ka64* = I: 8,075,488 A > T (L > Stop); *ka66* = I: 8,072,572 C > T (E > K); *ka200* = X: 16,544,813 C > T (Q > Stop); *ka201* = V: 13,150,224 AGG > AG frameshift; *ka202* = III: 13,676,892 G > A (Q > Stop); *ka203* = I: 8,077,873 G > A splice site acceptor change; *ka204* = X: 5,550,502 A > T (C > Stop); *m160* III: 13,686,367 G > A (R > Stop); *nx61* = X: 5,545,532–5,547,540 deletion; *p802* = IV: 3,797,722 G > A (Q > Stop); *p808* = X: uncharacterized; *p816* = X: uncharacterized ∼600-bp deletion.

Extensive EMS genetic screens for ivermectin- and abamectin-resistant mutants were carried out previously and mapping of 2 mutants identified 2 IFT-related mutants (*che-3*(*ka32*) and *dhc-3*(*ka33*)) (Page 2018). In this current study, a new forward genetic screening to identify moxidectin-resistant strains was performed. Together, these screens identified 31 mutants resistant to macrocyclic lactones, which also had their DiI dye-filling phenotypes characterized (Table 2). Based on phenotype, TP236(*ka30*), TP241(*ka35*), TP272(*ka64*), and TP274(*ka66*) from the previous abamectin screen (Page 2018) along with TP375(*ka200*), TP378(*ka201*), TP384(*ka202*), TP386(*ka203*), and TP388(*ka204*) from the current moxidectin screen were selected for backcrossing, whole-genome sequencing, and SNP mapping. Of the selected strains, all were resistant to ivermectin and moxidectin. TP388(*ka204*) was dye-filling positive while all others were dye-filling negative. The whole-genome sequencing and mapping data (aligned reads available at https://www.ncbi.nlm.nih.gov/sra/PRJNA768320) identified novel alleles of *osm-3*, *che-3* (4 different alleles), *osm-1*, *dhc-3*, *dyf-2*, and *ifta-1* (Fig. 2) as the causative genes for resistance to macrocyclic lactones. Details of identified alleles are listed in Supplementary Table 2.

**Table 2.** Resistance profiles and causative genes for resistance to macrocyclic lactones in EMS-generated mutant strains.

Strain (selection screen used for isolation)	Assigned allele	Causal gene	Mutation/effect	DYF	IVM R	MOX R
TP236 (10 nM ivermectin)	*ka30*	*osm-3*	Substitution/nonsense	−	+	+
TP241 (50 nM abamectin)	*ka35*	*che-3*	Deletion/coding	−	+	+
TP272 (10 nM ivermectin)	*ka64*	*che-3*	Substitution/nonsense	−	+	+
TP274 (10 nM ivermectin)	*ka66*	*che-3*	Substitution/missense	−	+	+
TP375 (10 nM moxidectin)	*ka200*	*osm-1*	Substitution/nonsense	−	+	+
TP378 (10 nM moxidectin)	*ka201*	*dhc-3*	Deletion/frameshift	−	+	+
TP384 (10 nM moxidectin)	*ka202*	*dyf-2*	Substitution/nonsense	−	+	+
TP386 (10 nM moxidectin)	*ka203*	*che-3*	Splice site substitution	−	+	+
TP388 (10 nM moxidectin)	*ka204*	*ifta-1*	Substitution/nonsense	+	+	+

Dyf, DiI amphid dye filling; IVM R, ivermectin resistance; MOX R, moxidectin resistance; +, dye filling/resistant; −, dye filling defective/susceptible.

## Discussion

### IFT protein resistances and redundancies

**Fig. 3.**
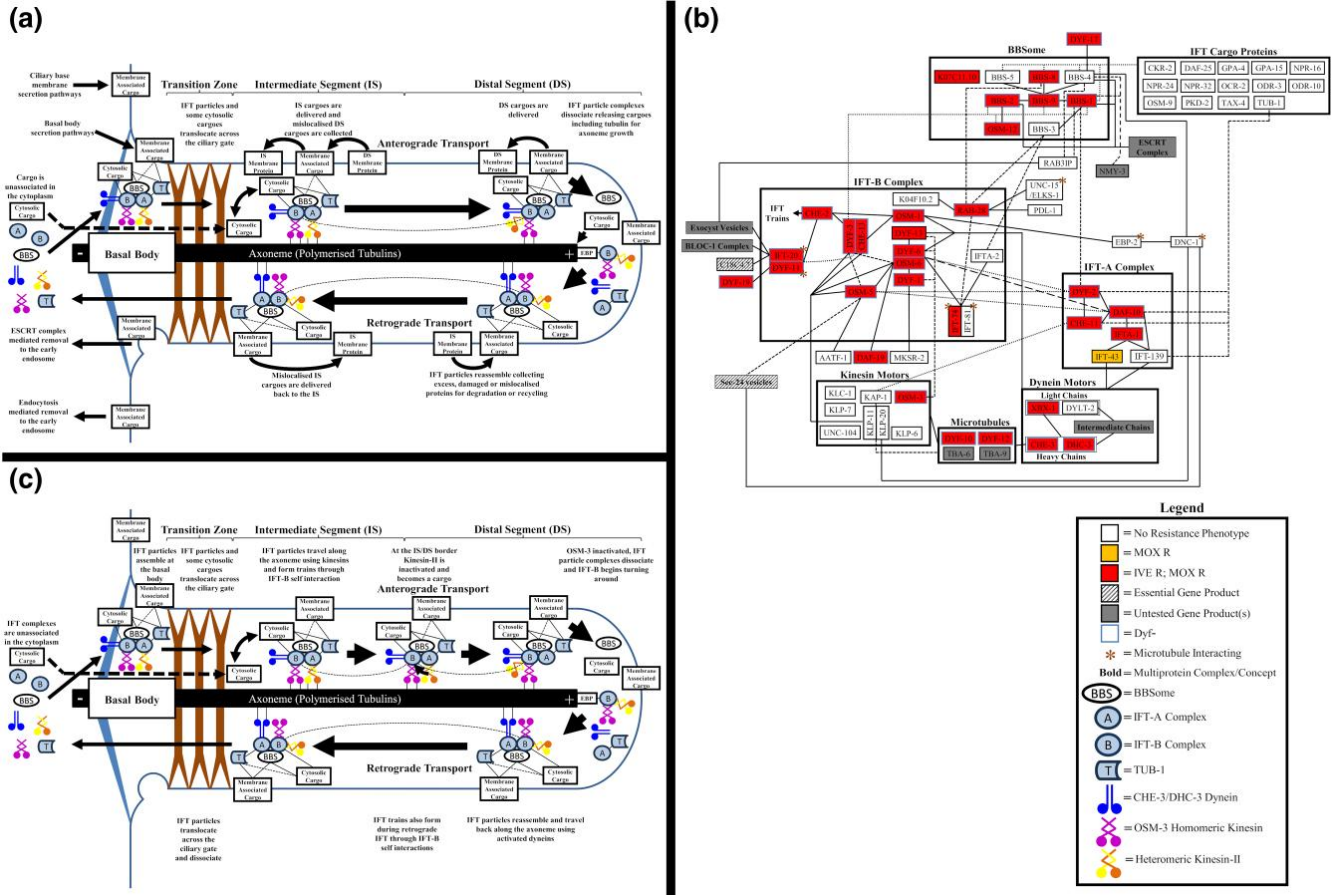
IFT in *C. elegans* and resistance patterns in the IFT protein–protein interaction network. a) Summary of ciliary cargo transport in *C. elegans* during IFT. Line = protein/complex–protein/complex interaction; small arrow = change in protein or complex localization or interaction; large arrow = direction of IFT particle travel. b) A simplified version of predicted IFT protein–protein interaction network in *C. elegans* showing resistances found in mutants of each node. Box = group of proteins from the same complex or with the same function; line = predicted protein/complex–protein/complex interaction; small arrow = protein self-interaction. c) Summary of IFT complex interactions during IFT *C. elegans*. Line = protein/complex–protein/complex interaction; small arrow = change in protein or complex localization or interaction; large arrow = direction of IFT particle travel.

The import and transport of ciliary proteins by IFT are highly conserved throughout Eukaryota (Fig. 3a) with defects impacting cell motility, migration, signaling, and division and the ability to sense environmental stimuli (Boehlke *et al*. 2015; Taschner and Lorentzen 2016; Prevo *et al*. 2017; Reiter and Leroux 2017). IFT mutations have also recently been linked to ivermectin resistance in nematodes (Dent *et al*. 2000; Urdaneta-Marquez *et al*. 2014; Page 2018). Many of the known intra and inter IFT particle complex protein–protein interactions (Haycraft *et al*. 2003; Behal *et al*. 2012; Kubo *et al*. 2016; Taschner and Lorentzen 2016; Klink *et al*. 2017; Woodsmith *et al*. 2017; Zhu *et al*. 2017; Funabashi *et al*. 2018; Taschner *et al*. 2018; Vuong *et al*. 2018; Nakayama and Katoh 2020) corresponded well with loss of function induced macrocyclic lactone resistance (Fig. 3b). This suggests that IFT particle core subunit structural interactions (BBS-1, BBS-2, BBS-9, CHE-11, DAF-10, DYF-1, DYF-2, DYF-3, DYF-6, IFT-74, IFTA-1, OSM-5, OSM-12, and RAB-28), tubulin import and tubulin microtubule interaction (DTF-10, DYF-11, IFT-20, and IFT-74), IFT train formation (IFT-80/CHE-2), and IFT particle turnaround (Ahmed *et al*. 2008; Williamson *et al*. 2012; Ishikawa *et al*. 2014; Yang and Huang 2020) (Fig. 3c) (DYF-6, DYF-13, and OSM-1) all play key roles in suppressing the macrocyclic lactone resistance mechanism. The importance of the BBSome cargo-interacting subunits (Su *et al*. 2014) (BBS-1 and BBS-8; Page 2018) implies the effector protein is probably a TUB-1-independent BBSome cargo. There is evidence from the resistance pattern observed in the homomeric kinesin (OSM-3) but not the heterotrimeric kinesin 2 (Snow *et al*. 2004; Prevo *et al*. 2015), ciliary dyneins (Signor *et al*. 1999; Wicks *et al*. 2000; Hao *et al*. 2011) (CHE-3, DHC-3, and XBX-1), and ciliary distal segment defect mutants (Burghoorn *et al*. 2007; Ou *et al*. 2007; Phirke *et al*. 2011; Olivier-Mason *et al*. 2013; Maurya *et al*. 2019) (*dyf-5*(*mn400*), *dyf-17*(*ox175*), *dyf-18*(*ok200*), *osta-1*(*tm5255*), *unc-101*(*sy108*), and *unc-119*(*e2498*)) that both functional anterograde and retrograde IFT (Fig. 3a) of the effector to the distal segment of the amphid cilia are required to exert an effect. The lack of a role for protein prenylation in macrocyclic lactone susceptibility implies that the resistance observed in *rab-28*(*ok3424*) is functioning by a loss in the BBS-8-dependent periciliary membrane interaction and not the BBS-3-mediated interaction with IFT. Resistance may therefore be the result of amphid pore defects (Jensen *et al*. 2016; Akella *et al*. 2019, 2020).

The use of single gene loss of function screens has some caveats in that proteins that have functional redundancy will give a greatly reduced phenotype. This was observed in protein dimers of the peripheral subunits of the IFT-B complex (IFT-20-DYF-11 and IFT-74-IFT-81) (Fig. 3b), which have known, but not always equal, redundancies (Kubo *et al*. 2016; Zhu *et al*. 2017). Similarly, the IFTA-1-interacting dynein-docking proteins IFT-139 and IFT-43 and the BBS-4 and BBS-5 cargo-interacting proteins of the BBSome are known to display redundancy when interacting with specific proteins (Behal *et al*. 2012; Xu *et al*. 2015; Yi *et al*. 2017; Scheidel and Blacque 2018), suggesting that future work should probe double mutants of these subunits to exclude their role in macrocyclic lactone resistance.

The nonredundant IFT particle subunits with no resistance phenotype association (BBS-3, DCT-14, IFTA-2, and K04F10.2) have niche roles in microtubule stability, receptor subpopulation trafficking, and cell signaling (Schafer *et al*. 2006; Pinkston-Gosse and Kenyon 2007; Li and Hu 2015; Sanders *et al*. 2015) suggesting that these processes and downstream effectors are nonessential for the resistance mechanism. Our results also support that DiI dye filling is less prone to disruption by ciliary impairment than the mechanism that induces resistance to macrocyclic lactones, as resistance was associated with the BBSome, *dyf-13*(*mn396*), *ift-74*(*ok2866*), and *ifta-1*(*nx61*) mutants without complete loss of dye-filling capability. Alternatively, these phenotypes could suggest branching in the mechanisms of dye filling and resistance at those subunits. It was surprising that *dyf-13*(*mn396*) was found to have no impairment in DiI uptake as that strain was used to first identify the locus with FITC and DiO dyes (Starich *et al*. 1995); however, Dio and DiI do not always share the same staining pattern (Hong *et al*. 2019).

### Secretion pathways used by ciliary proteins

**Fig. 4.**
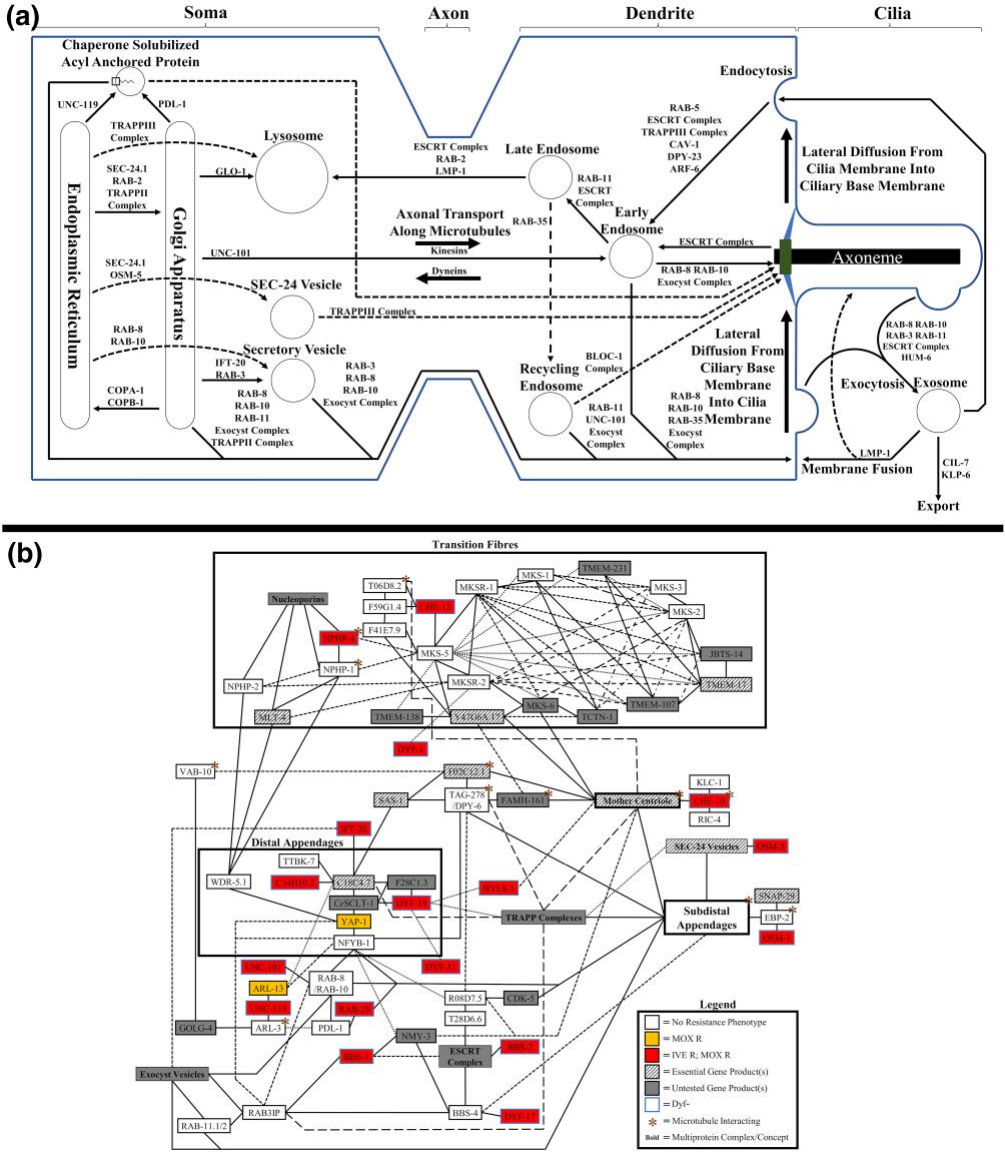
Ciliary protein trafficking pathways in *C. elegans* and resistance patterns in the ciliary gate protein–protein interaction network. a) Protein trafficking pathways used to deliver and remove ciliary proteins. Small arrow = show directionality of protein trafficking between cellular locations or organelles with key proteins and complexes involved in trafficking listed next to the arrow (placed before junctions if merging into a common secretion pathway); large arrow = directionality of axonal transport or passive diffusion. b) A simplified version of predicted basal body protein–protein interaction network in *C. elegans* showing resistances found in mutants of each node. Box = group of proteins from the same complex or with the same function; line = predicted protein–protein interaction; / = multiple (2–4) candidate genes with homology to a node found in other species (if gene IDs differ only by the last digit, then only the last digit is shown to the right of the candidate with a similar ID); *Ce* (node name of vertebrate ortholog) = multiple (>4) candidate genes with homology to the node found in other species.

Proteins produced in the soma of ciliated neurons, including those for ciliogenesis, IFT, and any downstream effectors for macrocyclic lactone susceptibility, require delivery to the ciliary gate using one of several secretory pathways (Sato *et al*. 2005–2018; Nachury *et al*. 2010; Ding *et al*. 2017; Monis *et al*. 2017; Mukhopadhyay *et al*. 2017; Leitch *et al*. 2014; Cromm *et al*. 2019) (summarized in Fig. 4a). The results suggest that the proteins that influence macrocyclic lactone resistance are being secreted via the UNC-101 and UNC-119 secretory pathways before transport along the axon using one or more unidentified axonal kinesins, whose direction of transport along polarized microtubules is dependent on UNC-33 and UNC-44 (Goldstein and Yang 2000; Muresan 2000; Maniar *et al*. 2011). There is an indication that the effector proteins, along with other proteins for cilia maintenance, are being loaded onto IFT particles that are forming in an ARL-13-dependent manner, making ARL-13 a candidate as one of the effectors of the UNC-119 secretory pathway (Ou *et al*. 2007; Zhang *et al*. 2016; Cromm *et al*. 2019). As some protein trafficking complexes have core subunits that are essential (making them difficult to probe directly), potential roles of the SEC-24(COPII), BLOC-1, and TRAPP complexes/pathways in macrocyclic lactone resistance cannot be entirely excluded. An alternative hypothesis for the resistance seen in *unc-33*(*e1193*) and *unc-44*(*e1197*) is that the marginally shorter cilia have a smaller area of membrane available for macrocyclic lactone interaction (Hedgecock *et al*. 1985).

### Elements of the ciliary gate important for resistance

IFT particles and other ciliary proteins cross the semi-impermeable ciliary gate (Lambacher *et al*. 2016; Li, Jensen, Park, *et al*. 2016; Garcia-Gonzalo and Reiter 2017; Endicott and Brueckner 2018; Blasius *et al*. 2019). They gain entry to the cilia via the basal body, through the interaction with several proteins from the basal body, distal and subdistal appendages, and protein trafficking complexes (Kilmartin 2003; Bowers *et al*. 2004; Yoshimura *et al*. 2007; Babbey *et al*. 2010; Kaplan *et al*. 2010; Zhao and Malicki 2011; Joo *et al*. 2013; Wei *et al*. 2013; Li, Chen, Fisher, *et al*. 2016; Mazo *et al*. 2016; Ojeda Naharros *et al*. 2017; Zhang *et al*. 2017) (simplified in Fig. 4b). There is evidence from the results that the effector for the macrocyclic lactone resistance phenotype is gaining entry to the cilia as part of IFT particles in a DYF-19, and potentially C18C4.7, dependent manner (Wei et al. 2013) with other subunits of the distal appendages (C14H10.2 and YAP-1) and linker(s) to the mother centriole (Dammermann *et al*. 2009; Wei *et al*. 2016) (HYLS-1) also having key roles. As *yap-1* is an ortholog of a Hippo pathway transcription factor (Iwasa *et al*. 2013; Lee *et al*. 2018) that also shares homology with the distal appendage subunit CEP164, there will be a need to dissect if resistance in mutants is occurring through the same mechanism as the other distal appendage proteins. The macrocyclic lactone resistance that was observed in the novel dye-filling defective (Dyf) phenotype-associated gene *c14h10.2*(*tm10737*) suggests that in addition to being a predicted CEP123 ortholog, it may interact with one or more of the IFT particle complexes.

The interactions between transition fiber proteins are highly redundant in *C. elegans* (Fig. 4b), meaning that defects in multiple proteins are required to cause the ciliary gate to become permeable, leading to ciliary defects and associated dye-filling phenotypes (Lambacher *et al*. 2016; Garcia-Gonzalo and Reiter 2017; Warburton-Pitt *et al*. 2012; Jensen *et al*. 2016). Consequently, only a single-transition fiber-encoding gene, *nphp-4*(*tm925*), was linked to resistance in this study. Among transition fiber-associated proteins and complexes, the TOGARAM1 ortholog CHE-12 has previously been associated with ivermectin resistance (Page 2018; Latour *et al*. 2019), so the lack of macrocyclic lactone resistance observed in mutants of the periphery subunits was surprising and suggests that unlike vertebrates there is either redundancy or that CHE-12 alone is sufficient for axoneme tubulin modification (Latour *et al*. 2019). Of the transition fiber-associated proteins, whose specific protein–protein interactions remain to be determined, the resistance observed in *osta-1*(*tm5255*) is potentially explained by reduced distal segment surface area (Olivier-Mason *et al*. 2013) resulting in impaired moxidectin uptake or tethering of cilium-sequestered transcription factors. The role of DYF-17 in distal segment assembly is currently unknown, but orthologs interact with BBS-4 (Lee *et al*. 2005; Phirke *et al*. 2011) suggesting a function in facilitating BBSome gating.

### Exosomes, recycling, and degradation pathways

Involvement of the UNC-101 (a clathrin adapter protein) secretion pathway and requirement of retrograde IFT for maintaining macrocyclic lactone susceptibility, along with IFT being associated with exosome release phenotypes (Nager *et al*. 2017; Akella *et al*. 2019), raised the possibility that endocytosis, secretory vesicles, trafficked endosomes, and the cycling of exosomes (all processes that require membrane folding and targeted fusion; Sato *et al*. 2005–2018; Mayor *et al*. 2014; Ni *et al*. 2020) could be involved in the macrocyclic lactone resistance mechanism. From the results, it can be deduced that the membrane folding is occurring via one or more of the clathrin-independent pathways (Mayor *et al*. 2014), with only the RAB-35-dependent fast endosome recycling pathway (Sato *et al*. 2008; Grant and Donaldson 2009) having a role in macrocyclic lactone resistance downstream of IFT. The lack of resistance observed in mutants for the other pathways (Supplementary Table 1) suggests that the slow endosome recycling pathway, lysosomal degradation pathway, and exosomes are not involved in anthelmintic resistance. Loss of RAB-35 might be causing resistance by changes in membrane protein and receptor populations, which could lead to upregulation of resistance gene expression, removal of effectors for anthelmintic uptake, or a restriction in primary target numbers, or by the additional roles that RAB-35 has in cell migration, neurite outgrowth, and cell polarity (Sato *et al*. 2008; Grant and Donaldson 2009; Overeem *et al*. 2015; Klinkert and Echard 2016).

### The gap junction mutants

Innexins form intercellular channels that function as gap junctions in neurotransmission and allow the exchange of small ions and compounds, including those for nucleotide signaling (Schumacher *et al*. 2012; Voelker *et al*. 2019), but might also facilitate the neural distribution of lipophilic dyes and anthelmintics. Comparison of the ivermectin resistance associated innexins *unc-7*(*e5*) and *unc-9*(*e101*), which are hypothesized to function by modulating the transmission of neurotoxic anthelmintic induced excitations (Dent *et al*. 2000), with *inx-19*(*ky634*) suggests that separate mechanisms are involved as the dye-filling defects indicate structural abnormalities of the ciliated amphid neurons. The macrocyclic lactone resistance and dye-filling phenotypes could be caused by the channel functions of INX-19 or the roles it has in determining neural cell fate (Chuang *et al*. 2007; Schumacher *et al*. 2012; Voelker *et al*. 2019), which could be important for the differentiation into cells involved in dye uptake and anthelmintic resistance.

### Whole-genome sequencing of forward genetic screen mutants

The causative genes identified by whole-genome sequencing of the macrocyclic lactone-resistant mutants were all found to be involved in IFT. This is not unexpected, as ciliogenesis and IFT are complex nonredundant processes requiring the interaction of multiple genes. This would make the many genes involved in IFT statistically more likely to undergo mutation than single downstream effectors that rely on functional cilia. The dynein heavy chains *che-3* and *dhc-3* were overrepresented in the forward genetic screens for macrocyclic lactone resistance, as has been found for screens probing dye-filling defects (Starich *et al*. 1995; Ou *et al*. 2007; Page 2018). This finding is linked to the fact that EMS-induced loss-of-function mutations are proportional to gene size (Gengyo-Ando and Mitani 2000), making the long-coding sequences of dynein heavy chains (12,516 and 9,828 nt for *che-3* and *dhc-3*, respectively) more prone to mutation than smaller IFT genes.

### Potential mechanisms of resistance

**Fig. 5.**
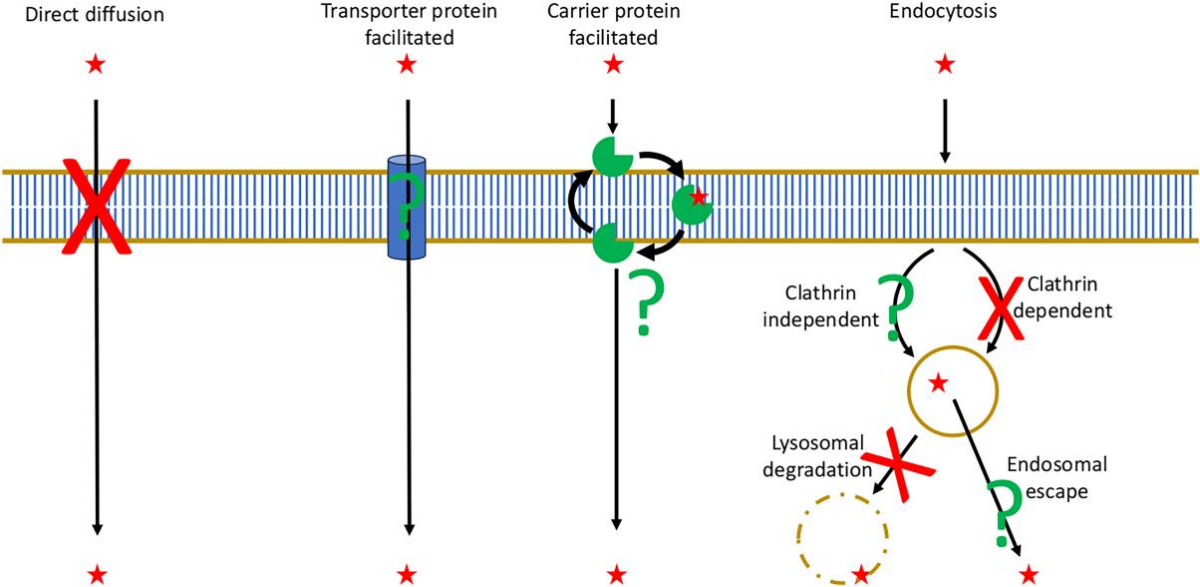
Potential routes of macrocyclic lactone entry into *C. elegans* tissues and possibilities eliminated. Summary of routes that would facilitate macrocyclic lactone entry into *C. elegans*. Red star = macrocyclic lactone molecule; small arrow = show directionality of transport; large arrows = carrier protein cycling between membrane surfaces; gold circle = endosome; red cross = possible entry route that has been eliminated; green question mark = potential entry route remaining.

The chemical properties of ivermectin and moxidectin prevent spontaneous crossing of cell membranes (Escher *et al*. 2008) meaning entry into organisms must be facilitated by either an extracellular membrane-associated carrier protein or transporter or by endocytic pathways (summarized in Fig. 5). The results suggest that the major routes of endocytosis and lysosomal degradation are not a significant mechanism for entry while a capacity for the compounds to induce endosomal escape has never been investigated. This means that uptake is most likely dependent on an elusive protein effector that may localize primarily to the amphid ciliary distal segments of ADL neurons.

There is ongoing debate as to whether resistance correlated with defective cilia (represented by *osm-3* mutants) is caused by reduced uptake (Dent *et al*. 2000; Urdaneta-Marquez *et al*. 2014; Page 2018) or by increased P-glycoprotein-facilitated export in the gut as part of an NHR-8-mediated intertissue signaling pathway (Guerrero *et al*. 2021). An *osm-3*;*nhr-8* double mutant was recently reported to have higher resistance than wild-type controls (Guerrero *et al*. 2021), indicating that additional resistance mechanisms are involved. The role of NHR-8 in the macrocyclic lactone-resistant mutants identified in this study could be further investigated using the P-glycoprotein inhibitor verapamil in combination with fluorescently labeled anthelmintics to observe effects on uptake.

## Conclusion

The findings of this study not only support strong evidence that the amphid cilia play an important role in responding to xenobiotic challenge by the macrocyclic compounds ivermectin and moxidectin (Page 2018) but also refine the location of the effectors to the distal segment of the cilia. The results from this study suggest that the effectors possess either a transmembrane domain or are anchored via a myristoyl or palmitoyl group. This study also uncovers the pathways used to deliver the effectors and other ciliary proteins in *C. elegans* and identifies C14H10.2 as a potential CEP123 ortholog. Due to the strong correlation between IFT function with dye-filling defects and resistance to macrocyclic lactones, it may be possible to use resistance phenotypes to identify if novel dye-filling mutants from forward genetic screens are upstream or downstream of IFT. If the resistance-causing genes uncovered in this study have the same functions in other nematode species, then there would be important implications for anthelmintic resistance-monitoring strategies.

## Supplementary Material

jkae009_Supplementary_Data

## Data Availability

The strains *dyf-17*(*ox175*) and *dyf-19*(*jhu455*) can be made available if unable to be obtained from their creators (Eric Jorgessen and Jinghua Hu, respectively). All other strains are available for purchase from the stock centers mentioned in the Methods section. Aligned reads are available at https://www.ncbi.nlm.nih.gov/sra/PRJNA768320. Images of dye-filling assays for individual strains are available upon request. All survival assay data are in the Supplementary Methods. Supplemental material available at G3 online.
