# Supplementary material for: The relationship between intraflagellar transport and upstream protein trafficking pathways and macrocyclic lactone resistance in Caenorhabditis elegans
Source: G3 (Bethesda). 2024 Jan 16;14(3):jkae009. doi: 10.1093/g3journal/jkae009 (PMC10917524; doi:10.1093/g3journal/jkae009)
Supplement: jkae009_Supplementary_Data [file jkae009_supplementary_data.zip › Table_S2_G3-2023-404796.docx]

**Table S2. Mutations near the resistance locus identified by whole genome sequencing in EMS derived macrocyclic lactone resistant mutants.** **Underline** = probable causative gene for resistance in the strain.

| **Mutant Strain** | **Gene Name/ID** | **Type Of Mutation** | **Genomic Position** |
| --- | --- | --- | --- |
| TP236(*ka30*) | *t23e1.1* | D/N codon change | IV: 3,154,565 |
|  | *otpl-1* | 1bp insertion frameshift | IV: 3,570,823 |
|  | *osm-3* | Q/Stop codon change | IV: 3,797,404 |
|  | *oac-23* | D/N codon change | IV: 3,882,822 |
|  | *f35f11.2* | 1bp insertion frameshift | IV: 4,040,399 |
|  | *f49f1.5* | C/W codon change | IV: 4,120,292 |
|  | *col-34* | V/E codon change | IV: 4,243,917 |
|  | *set-9* | S/N codon change | IV: 4,316,240 |
|  | *y24d9a.5* | A/V codon change | IV: 4,398,680 |
|  | *f29b9.1* | L/I codon change; E/Stop codon change | IV: 4,665,827; IV: 4,665,860 |
|  | *srz-24* | A/G splice acceptor change | IV: 4,980,410 |
|  | | | |
| TP241(*ka35*) | *pigo-1* | GATCATTTGATTGTAATTGGA inframe insertion; GATCTTTTGATTGTAATTGGA inframe insertion; D/N codon change | I: 6,066,245; I:6,066,247; I: 6,067,365 |
|  | *gpb-2* | Q/R codon change | I: 7,319,247 |
|  | *vh15n14r.1* | H/Q codon change | I: 7,782,452 |
|  | *k04g2.10*; *che-3* | 20,214 bp deletion | I: 8,058,869-8,079,083 |
|  | *t28b8.4* | G duplication splice donor change | I: 8,161,385 |
|  | | | |
| TP272(*ka64*) | *c46h11.7* | Y113 deletion; D110 deletion; D110T111/A deletion | I: 5,042,618; I: 5,042,624; I: 5,042,629 |
|  | *f48c1.9* | A/C codon change | I: 5,332,435 |
|  | *pigo-1* | GATCTTTTGATTGTAATTGGA inframe insertion | I: 6,066,247 |
|  | *t10e9.8* | S/N codon change | I: 6,540,898 |
|  | *m05b5.1* | G/D codon change | I: 7,178,937 |
|  | *gpb-2* | Q/R codon change | I: 7,319,247 |
|  | *vh15n14r.1* | H/Q codon change | I: 7,782,452 |
|  | *che-3* | L/Stop codon change | I: 8,075,488 |
|  | *t28b8.4* | G duplication splice donor change | I: 8,161,385 |
|  | | | |
| TP274(*ka66*) | *pigo-1* | GATCTTTTGATTGTAATTGGA  inframe insertion | I: 6,066,247 |
|  | *ttx-7* | G/E codon change + splice region variant | I: 7,301,663 |
|  | *gpb-2* | Q/R codon change | I: 7,319,247 |
|  | *f27d4.6* | S/F codon change | I: 7,737,397 |
|  | *vh15n14r.1* | H/Q codon change | I: 7,782,452 |
|  | *che-3* | E/K codon change | I: 8,072,572 |
|  | *t28b8.4* | G duplication splice donor change | I: 8,161,385 |
|  | *tbcd-1* | S/C codon change | I: 8,368,425 |
|  | *f36a2.14* | G/E codon change | I: 8,825,345 |
|  | | | |
| TP375(*ka200*) | *dyc-1* | S/Y codon change | X: 14,058,448 |
|  | *f28h6.t1* | Stop/Y codon change | X: 14,115,705 |
|  | *f28h6.6* | V/A codon change | X: 14,123,918 |
|  | *sym-4* | Q/H codon change | X: 14,169,390 |
|  | *k06g5.1* | L/R codon change | X: 14,214,987 |
|  | *rbc-1* | C duplication frame shift | X: 14,615,054 |
|  | *daf-6* | Q/K codon change | X: 14,891,748 |
|  | *y70d2a.1* | A/E codon change | X: 14,922,320 |
|  | *ntr-2* | H/P codon change; A/S codon change | X: 14,926,056; X: 14,926,099 |
|  | *c18b12.6* | D/A codon change | X: 15,006,413 |
|  | *t10b10.3* | D/Y codon change | X: 15,179,408 |
|  | *snt-7* | L/W codon change | X: 15,198,744 |
|  | *h03a11.2* | Q/H codon change | X: 15,227,130 |
|  | *tag-97* | A/E codon change | X: 15,377,657 |
|  | *lron-3* | I/S codon change | X: 15,547,779 |
|  | *dyn-1* | L/F codon change | X: 15,569,928 |
|  | *lin-15b* | I/S codon change | X: 15,726,181 |
|  | *y7a5a.7* | H/Q codon change | X: 15,802,989 |
|  | *csb-1* | N/H codon change;D/H codon change; N/A codon change | X: 15,863,974; X: 15,864,082; X: 15,865,678 |
|  | *let-2* | G/R codon change | X: 16,382,786 |
|  | *f38e9.t2* | C duplication Stop/L codon change + frame shift | X: 16,461,486 |
|  | *f38e9.1* | E/A codon change | X: 16,474,106 |
|  | *osm-1* | Q/Stop codon change | X: 16,544,813 |
|  | *c06g1.1* | T/I codon change | X: 16,635,523 |
|  | *f43b10.1* | E/D codon change | X: 16,661,328 |
|  | *igc-53* | premature start codon | X: 16,718,944 |
|  | *f35b3.3* | A/V codon change | X: 17,015,946 |
|  | *f35b3.4* | V/G codon change | X: 17,020,129 |
|  | *k09e3.2* | I/L codon change | X: 17,117,863 |
|  | *sid-3* | Q/P codon change | X: 17,191319 |
|  | *y40c7b.3* | S/N codon change | X: 17,259,503 |
|  | *c53c11.5* | K/N codon change | X: 17,335,708 |
|  | *f10d7.5* | A/E codon change | X: 17,384,339 |
|  | | | |
| TP378(*ka201*) | *dhc-3* | 1bp deletion frameshift | V: 13,150,224 |
|  | *avr-15* | P/L codon change | V: 13,499,482 |
|  | *c56a3.8* | E/K codon change | V: 13,561,914 |
|  | *h37a05.4* | 4bp insertion frame shift; 8bp deletion frameshift | V: 13,647,376; V: 13,647,378 |
|  | *clec-57* | G/R codon change | V: 14,472,276 |
|  | *c30g7.2* | C duplication frame shift | V: 14,938,766 |
|  | *str-45* | T/I codon change | V: 15,159,894 |
|  | *ech-1.1* | S/L codon change | V: 15,358,597 |
|  | *f57a10.2* | G/S codon change;S/Stop codon change | V: 15,766,718; V: 15,766,903 |
|  | *str-103* | S/T codon change | V: 16,118,131 |
|  | *t13f3.8* | K/N codon change | V: 16,272,222 |
|  | *srh-167* | I/V codon change | V: 16,334,475 |
|  | *f36d3.4* | G/S codon change | V: 16,517,019 |
|  | *srx-54* | G/E codon change | V: 16,976,467 |
|  | | | |
| TP384(*ka202*) | *cest-12* | A duplication frame shift | III: 12,103,364 |
|  | *ttm-1* | N/T codon change | III: 12,953,329 |
|  | *t03f6.6* | premature start codon | III: 13,380,306 |
|  | *f11f1.8* | L/F codon change | III: 13,397,844 |
|  | *mig-18* | A/T codon change | III: 13,403,753 |
|  | *dyf-2* | Q/Stop codon change | III: 13,676,892 |
|  | *tyr-2* | W/Stop codon change | III: 13,744,643 |
|  | | | |
| TP386(*ka203*) | *y92h12bl.4* | 1bp deletion frameshift | I: 1,382,293 |
|  | *linc-131* | A duplication splice donor change | I: 1,696,287 |
|  | *met-1* | Q/E codon change | I: 4,263,368 |
|  | *c46h11.7* | D110 deletion | I: 5,042,624 |
|  | *pigo-1* | TCCAATTACAATCAAAAGATC inframe insertion | I: 6,066,247 |
|  | *bag-1* | D/E codon change | I: 6,564,220 |
|  | *f13g3.12* | S/F codon change | I: 7,296,394 |
|  | *gpb-2* | Q/R codon change | I: 7,319,247 |
|  | *vh15n14r.1* | H/Q codon change | I: 7,782,452 |
|  | *jph-1* | A/T codon change | I: 7,948,195 |
|  | *che-3* | G/A splice acceptor change | I: 8,077,873 |
|  | *t28b8.4* | G duplication splice donor change | I: 8,161,385 |
|  | *hum-1* | E/K codon change | I: 8,848,672 |
|  | *f14b4.1* | N/K codon change | I: 9,268,868 |
|  | | | |
| TP388(*ka204*) | *c15c7.4* | C/R codon change | X: 3,146,996 |
|  | *f35a5.1* | C duplication frameshift; A duplication frameshift; G duplication frameshift | X: 3,811,374; X: 3,812,044; X: 3,812,046 |
|  | *acr-10* | A/G splice acceptor change; T duplication frameshift | X: 4,025,129; X: 4,025,179 |
|  | *coel-1* | L/I codon change | X: 4,265,965 |
|  | *asb-2* | K/N codon change | X: 4,470,957 |
|  | *ifa-4* | A duplication frameshift; 1bp insertion frame shift | X: 4,914,745; X: 4,914,748 |
|  | *pks-1* | C/Y codon change | X: 5,437,941 |
|  | *ifta-1* | C/Stop codon change | X: 5,550,502 |
|  | *t22b7.3* | D/N codon change | X: 5,649,411 |
|  | *gnrr-4* | A/V codon change | X: 5,738,941 |
|  | *pak-1* | A/V codon change | X: 6,047,488 |
|  | *c03b1.10* | 4bp insertion frameshift | X: 6,357,781 |
|  | *c03b1.1* | C duplication frameshift | X: 6,374,879 |
|  | *atg-2* | S/N codon change | X: 6,813,178 |
