# Supplementary material for: The relationship between intraflagellar transport and upstream protein trafficking pathways and macrocyclic lactone resistance in Caenorhabditis elegans
Source: G3 (Bethesda). 2024 Jan 16;14(3):jkae009. doi: 10.1093/g3journal/jkae009 (PMC10917524; doi:10.1093/g3journal/jkae009)
Supplement: jkae009_Supplementary_Data [file jkae009_supplementary_data.zip › Supplemental_Methods_G3-2023-404796.docx]

# The uptake of avermectins in *Caenorhabditis elegans* is dependent on Intra-Flagellar Transport and other protein trafficking pathways

Robert A. Brinzer^1^*, Alan D. Winter^1^ and Antony P. Page^1^*

**1** Institute of Biodiversity, Animal Health and Comparative Medicine, University of Glasgow, Scotland, UK

***** [robert.brinzer@glasgow.ac.uk](mailto:robert.brinzer@glasgow.ac.uk) (RAB); [tony.page@glasgow.ac.uk](mailto:tony.page@glasgow.ac.uk) (APP)

# Supplementary Methods:

## **Chemicals**

Chemicals were purchased from the following sources:

**BDH Laboratory Supplies**: KCl (101984L), MgSO_4_ (101514Y) and Na_2_EDTA (100935V)

**Biotium**: DiI (60010)

**Fisher Chemical**: Glycerol (G/0650/17) and NaOH (S/4920/53)

**Invitrogen**: dNTPs (55082, 55083, 55084 and 55085) and UltraPure agar (15510-027)

**Oxoid**: Tryptone (LP0042) and Yeast Extract (LP0021)

**MP Biochemicals**: K_2_HPO_4_ (191432) and Tris (152176)

**Promega**: MgCl (A351H)

**Scientific Laboratory Supplies**: Na_2_HPO_4_ (CHE1876)

**Sigma**: agar (05038), albendazole (A4673), CaCl2 (C3306), cholesterol (C8667), DMSO (D5879), ethanol (E7023), ethyl methanesulfonate (M0880), gelatin (G8150), isopropanol (24137), ivermectin (18898), KH_2_PO_4_ (P5379), levamisole (L9756), MgCl_2_ (M2670), moxidectin (33746), peptone (70178), sodium azide (71289), streptomycin sulfate (56501) and TWEEN-20 (P5927)

**VWR Chemicals**: acetic acid (20104.304) and NaCl (27810.295)

**Zeiss**: Immersol (518F)

**Cotton Tree**: Petroleum Jelly.

## ***C. elegans* Strain List**

| CB101(*e101*)  CB1017(*e1017*)  CB1033(*e1033*)  CB1066(*e1066*)  CB1124(*e1124*)  CB1126(*e1126*)  CB1193(*e1193*)  CB1197(*e1197*)  CB1201(*e1201*)  CB1265(*e1265*)  CB1292(*e1292*)  CB1377(*e1377*)  CB1387(*e1387*)  CB1402(*e1402*)  CB234(*e234*)  CB3323(*e1815*)  CB3329(*e1809*)  CB3330(*e1810*)  CB3332(*e1812*)  CB3474(*e1880*)  CB3687(*e1960*)  CB398(*e398*)  CB4845(*e2498*)  CB4856  CB5(*e5*)  CB5542(*e2762*)  CB713(*e713*)  CB78(*e78*)  CB840(*e840*)  CX10(*ky10*)  CX20(*ky20*)  CX2386(*ky31*)  CX3410(*ky225*)  CX4148(*ky13*)  CX4533(*ok132*)  CX4544(*ak47*)  CX6161(*ky634*)  DA1316(*ad1305;vu227;pk54*)  DH1230(*b1025*)  DR2386(*m362*)  DR86(*m86*)  DR96(*e911*)  EG175(*ox175*)  ET100(*ok200*)  EU1006(*or404*)  FF41(*e2310*)  HA1857(*tm2256*)  JT11069(*ok279*)  JT5244(*sa22*)  JY190(*yz6*)  LSC32(*tm3082*)  LX950(*vs137*)  MH1946(*ku266*)  ML514(*ok193*)  MT14984(*n4622*)  MT3641(*n1602*)  MT3645(*n1606*)  MT3664(*n1600*)  MT4810(*n2046*)  MT5300(*n2144*)  MX124(*nx61*)  MX52(*nx77*)  N2  NG57(*gm57*)  NL790(*pk381*)  NL797(*pk477*)  NM210(*y250*)  NM2686(*js805*)  NM547(*js21*)  OH13098(*ot75*)  OK814(*cu13*)  PR672(*p672*)  PR802(*p802*)  PR808(*p808*)  PR811(*p811*)  PR813(*p813*)  PR816(*p816*)  PS529(*sy108*)  PT8(*sy606*)  QR160(*vh22*)  RB1052(*ok999*)  RB1288(*ok1387*)  RB1304(*ok1417*)  RB1328(*ok1445*)  RB1330(*ok1447*)  RB1365(*ok1541*)  RB1374(*ok1559*)  RB1501(*ok1769*)  RB1550(*ok1863*)  RB1593(*ok1958*)  RB1600(*ok1972*)  RB1630(*ok2006*)  RB1667(*ok2065*)  RB1679(*ok2089*)  RB1827(*ok2365*)  RB1834(*ok2373*)  RB1875(*ok2425*)  RB1912(*ok2485*)  RB1928(*ok2523*)  RB1938(*ok2541*)  RB1951(*ok2565*)  RB1975(*ok2609*)  RB2002(*ok2647*)  RB2175(*ok2942*)  RB2256(*ok3053*)  RB2353(*ok3191*)  RB2375(*ok3228*)  RB2464(*ok3403*)  RB2484(*ok3424*)  RB2509(*ok3472*)  RB2514(*ok3482*)  RB2546(*ok3537*)  RB2555(*ok3560*)  RB2574(*ok3582*)  RB674(*ok406*)  RB743(*ok500*)  RB759(*ok525*)  RB820(*ok391*)  RB849(*ok676*)  RB906(*ok769*)  RM663(*ok417*)  RT206(*b1013*)  SD551(*ga89*)  SP1205(*mn335*)  SP1234(*m160*)  SP1603(*m185*)  SP1678(*mn396*)  SP1709(*e1383*)  SP1712(*m175*)  SP1713(*mn392*)  SP1735(*m537*)  SP1745(*mn400*)  TM10162(*tm10162*)  TM10579(*tm10579*)  TM10737(*tm10737*)  TM10972(*tm10972*)  TM11304(*tm11304*)  TM11397(*tm11397*)  TM11456(*tm11456*)  TM12030(*tm12030*)  TM12238(*tm12238*)  TM1357(*tm1357*)  TM1416(*tm1416*)  TM1447(*tm1447*)  TM1475(*tm1475*)  TM1503(*tm1503*)  TM1703(*tm1703*)  TM1745(*tm1745*)  TM1830(*tm1830*)  TM1892(*tm1892*)  TM2081(*tm2081*)  TM2159(*tm2159*)  TM2390(*tm2390*)  TM2452(*tm2452*)  TM2518(*tm2518*)  TM2526(*tm2526*)  TM2598(*tm2598*)  TM2705(*tm2705*)  TM3038(*tm3038*)  TM3067(*tm3067*)  TM3304(*tm3304*)  TM3611(*tm3611*)  TM3824(*tm3824*)  TM3920(*tm3920*)  TM4409(*tm4409*)  TM4830(*tm4830*)  TM4852(*tm4852*)  TM5061(*tm5061*)  TM5125(*tm5125*)  TM5255(*tm5255*)  TM5550(*tm5550*)  TM5848(*tm5848*)  TM592(*tm592*)  TM604(*tm604*)  TM6453(*tm6453*)  TM6588(*tm6588*)  TM6737(*tm6737*)  TM705(*tm705*)  TM7257(*tm7257*)  TM7884(*tm7884*)  TM8137(*tm8137*)  TM8587(*tm8587*)  TM8684(*tm8684*)  TM925(*tm925*)  TP236(*ka30*)  TP239(*ka33*)  TP241(*ka35*)  TP272(*ka64*)  TP274(*ka66*)  TP375(*ka200*)  TP378(*ka201*)  TP384(*ka202*)  TP386(*ka203*)  TP388(*ka204*)  TU253(*u253*)  VC1026(*ok1494*)  VC1062(*gk471*)  VC1066(*gk456*)  VC1116(*ok1538*)  VC1130(*gk508*)  VC117(*gk45*)  VC1179(*gk545*)  VC1188(*gk546*)  VC1228(*tm324*)  VC1262(*ok1677*)  VC1268(*gk567*)  VC1312(*gk512*)  VC1316(*gk537*)  VC1428(*gk653*)  VC1569(*ok2053*)  VC1579(*gk737*)  VC1580(*gk738*)  VC1625(*gk762*)  VC1977(*ok2142*)  VC2129(*ok2681*)  VC2140(*ok2866*)  VC2343(*gk1232*)  VC2361(*ok3079*)  VC2421(*ok3192*)  VC282(*gk157*)  VC3015(*ok3707*)  VC3113(*ok3771*)  VC3240(*gk3188*)  VC3981(*gk5058*)  VC637(*ok998*)  VC709(*gk312*)  VC837(*ok1111*)  VC934(*gk382*)  VC947(*gk427*)  YH461(*tm1724*)  ZP541(*jhu455*)  ZZ1(*x1*)  ZZ15(*x15*) |  |  |  |  |  |  |  |  |
| --- | --- | --- | --- | --- | --- | --- | --- | --- |

|  |  |  |  |  |
| --- | --- | --- | --- | --- |
|  |  |  |  |  |

## **Albendazole and levamisole resistance assays**

Anthelmintic stock solutions were prepared as follows: 50mM albendazole stock was made by dissolving in DMSO at 31°C with vigorous agitation; 1M levamisole stock was made by dissolving in sterile distilled water. Stock solutions were dispensed into 1ml aliquots and stored at -20°C.

Albendazole and levamisole resistance was determined using uncoordinated phenotype assays by picking 5 adult worms of the strain to be tested onto each plate with two biological and two technical replicates. At day 3 and day 6 a random sample of 20 worms per plate were poked on their head with a platinum wire and scored for the ability to reverse backwards (an inability to reverse corresponds to an Unc or uncoordinated phenotype). For mutant strains which innately show an uncoordinated phenotype (Unc), a different scoring criteria was used; with worms being scored as resistant if any muscle movement was shown in response to being poked and scored as sensitive if they were completely paralysed. N2 was used as a negative (sensitive) control and CB3474(e1880) (for albendazole resistance), ZZ1(x1) or ZZ15(x15) (for levamisole resistance) were used as positive controls. Strains were considered resistant if over 50% of sampled worms were unaffected by the anthelmintic; categorised as moderately resistant (+) at the lower dose and strongly resistant (++) at the higher dose. If at the higher dose a strain was 100% unaffected it was classed as extremely resistant (+++). Susceptible strains had less than 50% of the sampled population unaffected at the lower dose (-).

## **Post Mutagenesis Worm Handling, Back Crossing and Data Processing Pipeline Details**

After EMS mutagenesis, from each of the three plates 20 individual adults were picked onto separate 9 cm NGM plates and populations allowed to develop to F2 adults. Populations were then bleached and the resulting F3 eggs transferred to 10 nM moxidectin containing NGM plates to select for resistance. An individual L4 hermaphrodite from each surviving plate was then propagated as a separate mutant line.

Hermaphrodites from a mutant line were backcrossed to males from the SNP rich Hawaiian strain CB4856 before performing single worm PCR (F1: GGGATCACCATATTTGGTAAGA, F2: CGAGTAATGCTTCAGACAAGT, R1: CATCGTGATGAAAAGTTGATGAC, lysis: 65°C for 60 mins then 95°C for 15 min, PCR: 20 cycles melting 1 min at 92°C, annealing 1 min at 56°C and extension 1 min at 72°C) on F1s which had laid eggs on separate plates to select for hybrids. The F2 L4 progeny of hybrids were then individually picked onto separate 3 cm NGM plates containing 10 nM moxidectin (TP375(*ka200*), TP378(*ka201*), TP384(*ka202*), TP386(*ka203*), TP387 and TP388(*ka204*) derived worms) or ivermectin (TP236(*ka30*), TP241(*ka35*), TP272(*ka64*) and TP274(*ka66*) derived worms)(total 250-300 plates). After 11-14 days the resistant populations were chunked onto NGM plates to recover for a generation before picking 20 F4 L4 worms from each population onto a 5 cm NGM plate containing 10 nM moxidectin or ivermectin. Overgrown plates from each population were washed with M9, pooled (approx. 60 total) and centrifuged at 1150g for 3 mins before performing 3 washes with M9 followed by gentle agitation for 2 hours to clear the gut of bacteria, 2 more M9 washes and removing excess supernatant after a final centrifugation. Samples were stored at -80°C for less than a month before performing genomic DNA extraction. Genomic DNA was stored at 4°C and dispatched for sequencing within 24 hours.

The NGS data was analysed and SNP-based mapping performed using MiModD tools on the Galaxy platform ^1^. The pipeline consisted of removing adapter sequences on the raw reads with the Cutadapt tool set to use Paired-end reads before alignment against a *C. elegans* reference genome sequence (WS220.64_chr.fa)(https://usegalaxy.org/u/gm2123/h/cloudmapot266proofofprinciple-with-unhidden-data) using the Map with BWA-MEM tool. The output BAM format file was put through the MiModD Reheader tool to change the sample name before performing variant calling using the MiModD Variant Calling tool with WS220.64_chr.fa used for the reference genome. The resulting bcf format file was used as the input for the MiModD Extract Variant Sites tool using HA_SNPS_Filtered_103346Variants_WS220.vcf (<https://usegalaxy.org/u/gm2123/h/cloudmapot266proofofprinciple-with-unhidden-data>) as the independently generated vcf data set. Linkage maps were then generated using the MiModD NacreousMap tool to visualize the genomic region of interest before using MiModD Rebase Sites to realign the vcf file to a more modern annotation of the *C. elegans* genome having ce10ToCe11.over.chain.gz (http://hgdownload.soe.ucsc.edu/goldenPath/ce10/liftOver/) as the input chain file. The realigned vcf file had the variants annotated using the SnpEff eff tool with SnpEff4.3 WBCel235.86 from the SnpEff download tool as the genome source before MiModD VCF Filter was applied to focus on the genomic region of interest which was then visualized with the MiModD Report Variants tool.

# References

1. Jalili V, Afgan E, Gu Q, Clements D, Blankenberg D, Goecks J, Taylor J, Nekrutenko A. 2020. The galaxy platform for accessible, reproducible and collaborative biomedical analyses: 2020 update. Nucleic Acids Res. 48(W1):W395-w402.
