# Supplementary material for: The relationship between intraflagellar transport and upstream protein trafficking pathways and macrocyclic lactone resistance in Caenorhabditis elegans
Source: G3 (Bethesda). 2024 Jan 16;14(3):jkae009. doi: 10.1093/g3journal/jkae009 (PMC10917524; doi:10.1093/g3journal/jkae009)
Supplement: jkae009_Supplementary_Data [file jkae009_supplementary_data.zip › Table_S1_G3-2023-404796.docx]

**Table S1. *C. elegans* mutants tested for ivermectin, moxidectin, albendazole and levamisole resistance.**

**Underline** = previously identified mutants that are resistant to one of the anthelmintics tested. **Dyf** = DiI amphid dye filling; **IVM R** = Ivermectin resistance; **MOX R** = Moxidectin resistance; **+** = Dye filling/Resistant; **-** = Dye filling defective/Susceptible. **IFT** = intraflagellar transport component homology.

| **Gene (homology)** | **Strain (allele)** | **Mutation/effect** | **DYF** | **IVM R** | **MOX R** |
| --- | --- | --- | --- | --- | --- |
| **Transcription Factor Mutants** | | | | | |
| *aatf-1* (AATF ortholog) | TM5125(*tm5125*) | Deletion/Coding | + | - | - |
| *che-1* (zinc finger transcription factor) | PR672(*p672*) | Substitution/Nonsense | + | - | - |
| *che-1* (zinc finger transcription factor) | OH13098(*ot75*) | Substitution/Nonsense | + | - | - |
| *daf-19* (RFX transcription factor) | DR86(*m86*) | Substitution/Nonsense | - | + | + |
| *hlh-4* (achaete-scute transcription factor) | TM604(*tm604*) | Deletion/Coding | - | + | + |
|  | | | | | |
| **Cell Migration/Adhesion Defect Mutants** | | | | | |
| *dyf-7* (ZP protein) | SP1735(*m537*) | Unknown | - | + | + |
| *mec-1* (EGF/Kunitz) | CB1066(*e1066*) | Splice site substitution | + | - | - |
| *mec-1* (EGF/Kunitz) | CB1292(*e1292*) | Substitution/Nonsense | + | - | - |
| *mec-8* (RRM domain/splice factor) | CB398(*e398*) | Substitution/Nonsense | + | + | + |
| *unc-6* (laminin) | CB78(*e78*) | Substitution/Missense | + | - | - |
| *unc-76* (FEZ1/FEZ2 ortholog) | DR96(*e911*) | Deletion/Frameshift | + | - | - |
| *vab-10* (dystonin ortholog/spectraplakin) | VC117(*gk45*) | Deletion/Coding | + | - | - |
|  | | | | | |
| **Amphid Channel Morphology Mutants** | | | | | |
| *che-6* (cyclin channel) | CB1126(*e1126*) | Unknown | + | - | - |
| *daf-6* (PTCHD-1/4 ortholog) | CB1377(*e1377*) | Substitution/Nonsense | - | + | + |
|  | | | | | |
| **Protein Secretion/Trafficking Defect Mutants** | | | | | |
| *aex-4* (SNAP23 ortholog, syntaxin) | JT5244(*sa22*) | Unknown | + | - | - |
| *arf-6* (ARF6 ortholog) | TM1447(*tm1447*) | Deletion/Coding | + | - | - |
| *arl-3* (ARL3 ortholog) | TM1703(*tm1703*) | Deletion/Coding | + | - | - |
| *arl-13* (ARL13B ortholog) | TM1745(*tm1745*) | Deletion/Coding | + | - | + |
| *blos-1* (BLOC1S1 ortholog, BLOC-1 complex) | VC3015(*ok3707*) | Deletion/Coding | + | - | - |
| *cav-1* (caveolin ortholog) | RB1679(*ok2089*) | Insertion/Coding | + | - | - |
| *chc-1* (clathrin heavy chain ortholog) | DH1230(*b1025*) | Deletion/Coding | + | - | - |
| *che-14* (DISP1 ortholog) | CB3687(*e1960*) | Splice site substitution | - | - | - |
| *che-14* (DISP1 ortholog) | ML514(*ok193*) | Deletion/Coding | - | - | - |
| *dnc-1* (p150(glued) ortholog) | EU1006(*or404*) | Substitution/Missense | + | - | - |
| *dpy-23* (adapter related protein complex 2 subunit mu 1) | CB840(*e840*) | Deletion/Coding | + | - | - |
| *dyf-5* (map kinase) | SP1745(*mn400*) | Substitution/Nonsense | - | + | + |
| *dyf-18* (CDK-8/19/20 ortholog) | ET100(*ok200*) | Deletion/Coding | - | - | + |
| *exoc-7* (EXOC7 ortholog, exocyst complex) | RB1630(*ok2006*) | Deletion/Coding | + | - | - |
| *exoc-8* (EXOC8 ortholog, exocyst complex) | RB1928(*ok2523*) | Deletion/Coding | + | - | - |
| *fce-1* (ZMPSTE24 ortholog, FACE family) | TM2390(*tm2390*) | Deletion/Coding | + | - | - |
| *fce-2* (RCE-1 ortholog, FACE family) | TM2598(*tm2598*) | Deletion/Coding | + | - | - |
| *glo-2* (BLOC-1 complex) | TM592(*tm592*) | Deletion/Coding | + | - | - |
| *lmp-1* (LAMP ortholog) | RB2375(*ok3228*) | Deletion/Coding | + | - | - |
| *mvb-12* (MVB12A ortholog, ESCRT I Complex) | RB2514(*ok3482*) | Deletion/Coding | + | - | - |
| *odr-8* (UFSP2 peptidase ortholog) | CX2386(*ky31*) | Substitution/Missense | + | - | - |
| *osta-1* (SLC51A ortholog) | TM5255(*tm5255*) | Deletion/Coding | + | - | + |
| *pamn-1* (PAM ortholog) | VC2129(*ok2681*) | Deletion/Coding | + | - | - |
| *pdl-1* (phosphodiesterase 6D) | VC282(*gk157*) | Insertion/Coding | + | - | - |
| *rab-2* (RAB family) | CB713(*e713*) | Substitution/Missense | + | - | - |
| *rab-3* (RAB family | NM210(*y250*) | Substitution/Nonsense | + | - | - |
| *rab-8* (RAB family) | TM2526(*tm2526*) | Deletion/Coding | + | - | - |
| *rab-10* (RAB family) | VC1026(*ok1494*) | Insertion/Deletion | + | - | - |
| *rab-11.1* (RAB11B ortholog, RAB family) | TM2159(*tm2159*) | Deletion/Coding | + | - | - |
| *rab-11.2* (RAB11A ortholog, RAB family) | TM2081(*tm2081*) | Deletion/Coding | + | - | - |
| *rab-35* (RAB family) | RT206(*b1013*) | Substitution/Nonsense | + | + | + |
| *rab3ip* (RAB3A interacting protein ortholog) | TM2518(*tm2518*) | Deletion/Coding | + | - | - |
| *ric-4* (SNAP25 ortholog, syntaxin) | VC709(*gk312*) | Deletion/Coding | + | - | - |
| *rpi-2* (RP2 ortholog) | RB1550(*ok1863*) | Insertion | + | - | - |
| *sec-6* (EXOC3 ortholog, exocyst complex) | TM8684(*tm8684*) | Deletion/Coding | + | - | - |
| *sec-22* (SEC22B ortholog, SNAP receptor) | RB2256(*ok3053*) | Deletion/Coding | + | - | - |
| *sedl-1* (TRAPPC2 ortholog) | RB1912(*ok2485*) | Unknown | + | - | - |
| *snpn-1*(SNAPIN ortholog, BLOC-1 complex) | TM1892(*tm1892*) | Deletion/Coding | + | - | - |
| *stam-1* (STAM ortholog, ESCRT-0 complex) | RB674(*ok406*) | Deletion/Coding | + | - | - |
| *t06g6.3* (myosin heavy chain like) | VC1188(*gk546*) | Deletion/Coding | + | - | - |
| *tsg-101* (TSG101 ortholog, ESCRT-I complex) | TM705(*tm705*) | Deletion/Coding | + | - | - |
| *trpp-10* (TRAPPC10 ortholog) | TM11397(*tm11397*) | Deletion/Coding | + | - | - |
| *unc-18* (syntaxin) | CB234(*e234*) | Substitution/Nonsense | + | - | - |
| *unc-33* (CRMP1 ortholog, filamin binding) | CB1193(*e1193*) | Unknown | + | + | + |
| *unc-44* (ANK2/ANK3 ortholog) | CB1197(*e1197*) | Unknown | - | + | + |
| *unc-64* (syntaxin) | NM547(*js21*) | Substitution/Missense | + | - | - |
| *unc-101* (AP1M1 ortholog) | PS529(*sy108*) | Insertion | - | + | + |
| *unc-119* (HRG4 ortholog) | CB4845(*e2498*) | Insertion | - | + | + |
| *vamp-7* (VAMP8 ortholog, SNAP receptor) | TM6588(*tm6588*) | Deletion/Coding | + | - | - |
| *vps-20* (CHMP6 ortholog, ESCRT III complex) | RB1875(*ok2425*) | Insertion | + | - | - |
| *vps-36* (VPS36 ortholog, ESCRT II complex) | VC947(*gk427*) | Deletion/Coding | + | - | - |
| *vps-37* (VPS37C ortholog, ESCRT I complex) | VC1116(*ok1538*) | Deletion/Coding | + | - | - |
| *wdr-5.1* (has AHI1 homology, WDR5 ortholog) | RB1304(*ok1417*) | Deletion/Coding | + | - | - |
|  | | | | | |
| **Cilia Nucleation and Region Identity Mutants** | | | | | |
| *b0432.8* (TATDN3 ortholog, has FAM92 homology) | TM6737(*tm6737*) | Deletion/Coding | + | - | - |
| *c14h10.2* (JAKMIP3 ortholog, putative CEP123 ortholog) | TM10737(*tm10737*) | Deletion/Coding | - | + | + |
| *c26c6.8* (Carom ortholog) | TM3920(*tm3920*) | Deletion/Coding | + | - | - |
| *che-10* (rootelin, IFT) | CB3329(*e1809*) | Unknown | - | + | + |
| *che-12* (TOGARAM1 ortholog, IFT) | CB3332(*e1812*) | Splice site substitution | - | + | + |
| *dpy-6* (putative OFD1 ortholog) | CB5542(*e2762*) | Deletion/Coding | + | - | - |
| *dsh-1* (DVL2/DVL3 ortholog) | RB1328(*ok1445*) | Deletion/Coding | + | - | - |
| *dyf-17* (MAGEL2 like) | EG175(*ox175*) | Insertion/Coding | - | + | + |
| *dyf-19* (FBF1 ortholog) | TM12238(*tm12238*) | Deletion/Coding | + | - | - |
| *dyf-19* (FBF1 ortholog) | ZP541(*jhu455*) | Substitution/Nonsense | - | + | + |
| *ebp-1* (EB1/EB2/EB3 ortholog) | TM1357(*tm1357*) | Deletion/Coding | + | - | - |
| *ebp-2* (EB1/EB2/EB3 ortholog) | VC1579(*gk737*) | Deletion/Coding | + | - | - |
| *ebp-3* (EB1/EB2/EB3 ortholog) | VC637(*ok998*) | Deletion/Coding | + | - | - |
| *elks-1* (ERC2 ortholog, has ODF2/Cenexin homology) | NM2686(*js805*) | Unknown Deletion | + | - | - |
| *epi-1* (LAMA5 ortholog, has CEP128 homology) | NG57(*gm57*) | Unknown | + | - | - |
| *f41e7.9* (IVL/CSPP1 ortholog) | TM10972(*tm10972*) | Deletion/Coding | + | - | - |
| *f59g1.4* (ARMC9/JBTS-30 ortholog) | VC3981(*gk5058*) | Deletion/Coding | + | - | - |
| *frm-8* (has centriolin homology, FRMPD1 ortholog) | RB1501(*ok1769*) | Deletion/Coding | + | - | - |
| *gasr-8* (GAS8 ortholog) | VC2343(*gk1232*) | Deletion/Coding | + | - | - |
| *hum-6* (MYO7A ortholog, has SCLT1 homology) | RB1827(*ok2365*) | Deletion/Coding | + | - | - |
| *hyls-1* (hydrolethalus syndrome ortholog) | TM3067(*tm3067*) | Deletion/Coding | - | + | + |
| *k10g6.4* (ANKRD26 like) | VC1268(*gk567*) | Deletion/Coding | + | - | - |
| *mks-1* (MKS transition zone complex subunit 1) | TM2705(*tm2705*) | Deletion/Coding | + | - | - |
| *mks-2* (TMEM216 ortholog) | TM5061(*tm5061*) | Deletion/Coding | + | - | - |
| *mks-3* (TMEM67 ortholog) | VC1977(*ok2142*) | Deletion/Coding | + | - | - |
| *mks-5* (RPGRIP1L ortholog) | RB2574(*ok3582*) | Deletion/Coding | + | - | - |
| *mksr-1* (B9D1 ortholog) | VC1580(*gk738*) | Insertion | + | - | - |
| *mksr-2* (B9D2 ortholog) | TM2452(*tm2452*) | Deletion/Coding | + | - | - |
| *nfyb-1* (putative CBY1 ortholog) | OK814(*cu13*) | Insertion | + | - | - |
| *nphp-1* (nephrocystin 1 ortholog) | RB743(*ok500*) | Deletion/Coding | + | - | - |
| *nphp-2* (nephronophthisis homolog) | VC1428(*gk653*) | Deletion/Coding | + | - | - |
| *nphp-4* (nephrocystin 4 ortholog) | TM925(*tm925*) | Deletion/Coding | - | + | + |
| *npp-26* (nucleoporin) | TM10579(*tm10579*) | Deletion/Coding | + | - | - |
| *pals-8* (putative CEP131 ortholog) | TM12030(*tm12030*) | Deletion/Coding | + | - | - |
| *prmn-1* (PROM1 ortholog) | VC1179(*gk545*) | Deletion/Coding | + | - | - |
| *r08d7.5* (putative centrin ortholog) | TM3611(*tm3611*) | Deletion/Coding | + | - | - |
| *t04f8.6* (ninein ortholog) | TM4830(*tm4830*) | Deletion/Coding | + | - | - |
| *t06d8.2* (JBTS-25 ortholog) | TM3824(*tm3824*) | Deletion/Coding | + | - | - |
| *t28d6.6* (putative SFI1/DRG1 ortholog) | TM5550(*tm5550*) | Deletion/Coding | + | - | - |
| *tag-278* (putative OFD1 ortholog) | VC934(*gk382*) | Insertion | - | - | - |
| *ttbk-7* (TTBK2 ortholog) | TM4852(*tm4852*) | Deletion/Coding | + | - | - |
| *unc-15* (has ODF2/Cenexin homology, myosin heavy chain) | CB1402(*e1402*) | Substitution/Nonsense | + | - | - |
| *unc-54* (has SCLT1 homology, myosin heavy chain) | CB1201(*e1201*) | Unknown | + | - | - |
| *y6b3b.4* (SCAPER ortholog) | TM11456(*tm11456*) | Deletion/Coding | + | - | - |
| *yap-1* (WWTR1 ortholog, has CEP164 homology) | TM1416(*tm1416*) | Deletion/Coding | + | - | + |
|  | | | | | |
| **Microtubule Mutants** | | | | | |
| *ben-1* (β-tubulin homolog) | CB3474(*e1880*) | Unknown | + | - | - |
| *dyf-10* (α-tubulin homolog) | SP1709(*e1383*) | Unknown | - | + | + |
|  | | | | | |
| **Dynein and Kinesin Motor Mutants** | | | | | |
| *bmk-1* (kinesin, KIF11 ortholog) | RB820(*ok391*) | Deletion/Coding | + | - | - |
| *che-3* (Dynein HC avr-1, IFT) | CB1124(*e1124*) | Substitution/Nonsense | - | + | + |
| *dhc-1* (Dynein, DYNC1H1 ortholog) | QR160(*vh22*) | Substitution/Missense | + | - | - |
| *dhc-3* (Dynein HC, IFT) | TP239(*ka33*) | Insertion/ frameshift | - | + | + |
| *dli-1* (Dynein LC) | MH1946(*ku266*) | Substitution/Nonsense | + | - | - |
| *dylt-1* (Dynein LC, Tctex-1 family) | RM663(*ok417*) | Deletion/Coding | + | - | - |
| *dylt-2* (Dynein LC homolog) | VC1625(*gk762*) | Deletion/Coding | + | - | - |
| *kap-1* (KIFAP3 ortholog) | RB849(*ok676*) | Insertion/Coding | + | - | - |
| *klc-1* (Kinesin LC family) | RB1975(*ok2609*) | Deletion/Coding | + | - | - |
| *klp-4* (kinesin-13A family) | RB2546(*ok3537*) | Deletion/Coding | + | - | - |
| *klp-6* (kinesin-3 family) | TM8587(*tm8587*) | Deletion/Coding | + | - | - |
| *klp-7* (KIF2A ortholog) | TM7884(*tm7884*) | Deletion/Coding | + | - | - |
| *klp-8* (GCC1 ortholog) | RB1951(*ok2565*) | Insertion | + | - | - |
| *klp-11* (KIF3B/C ortholog) | VC1228(*tm324*) | Deletion/Coding | + | - | - |
| *klp-15* (kinesin like protein) | RB1593(*ok1958*) | Insertion/Deletion | + | - | - |
| *klp-20* (KIF3A ortholog) | RB2175(*ok2942*) | Deletion/Coding | + | - | - |
| *osm-3* (kinesin family, IFT) | PR802(*p802*) | Substitution/Nonsense | - | + | + |
| *unc-104* (KIF1A/B ortholog) | CB1265(*e1265*) | Substitution/Missense | + | - | - |
| *unc-116* (KIF5A/B/C ortholog) | FF41(*e2310*) | Insertion/Coding | + | - | - |
| *vab-8* (KIF26A ortholog) | CB1017(*e1017*) | Substitution/Nonsense | + | - | - |
| *wdr-60* (WDR60 ortholog) | TM6453(*tm6453*) | Insertion/Coding | + | - | - |
| *xbx-1* (DYNC2LI1 ortholog) | JT11069(*ok279*) | Deletion/Coding | - | + | + |
|  | | | | | |
| **IFT-A Complex Mutants** | | | | | |
| *che-11* (IFT140 homolog, IFT) | CB3330(*e1810*) | Substitution/Nonsense | - | + | + |
| *daf-10* (IFT122A homolog, WD repeat, IFT) | CB1387(*e1387*) | Substitution/Nonsense | - | + | + |
| *dyf-2* (IFT144 homolog, WRD19, IFT) | SP1234(*m160*) | Substitution/Nonsense | - | + | + |
| *ift-43* (IFT43 homolog, IFT) | TM8137(*tm8137*) | Insertion/Coding | + | - | + |
| *ifta-1* (IFT122B homolog, WDR35, IFT) | MX124(*nx61*) | Deletion/Coding | - | + | + |
| *ift-139* (IFT139 homolog) | VC1130(*gk508*) | Deletion/Coding | + | - | - |
|  | | | | | |
| **IFT-B Complex Mutants** | | | | | |
| *che-2* (IFT80 homolog, G-protein, WD repeat) | CB1033(*e1033*) | Unknown | - | + | + |
| *che-13* (IFT57/Hippi) | CB3323(*e1815*) | Substitution/Nonsense | - | + | + |
| *dyf-1* (IFT70 homolog, IFT) | SP1205(*mn335*) | Insertion/Frameshift | - | + | + |
| *dyf-3* (IFT38 homolog, CLUAP protein, IFT) | SP1603(*m185*) | Substitution/Nonsense | - | + | + |
| *dyf-6* (IFT46 homolog, IFT) | SP1712(*m175*) | Substitution/Nonsense | - | + | + |
| *dyf-11* (IFT54 homolog, IFT) | SP1713(*mn392*) | Substitution/Nonsense | - | + | + |
| *dyf-13* (IFT56 homolog, IFT) | SP1678(*mn396*) | Splice site substitution | + | + | + |
| *ift-20* (IFT20 homolog, IFT) | RB2353(*ok3191*) | Deletion/Coding | - | + | + |
| *ift-74* (IFT72/74 homolog, IFT) | VC2140(*ok2866*) | Deletion/Coding | + | + | + |
| *ift-81* (IFT81 homolog, IFT) | VC1312(*gk512*) | Deletion/Coding | + | - | - |
| *ifta-2* (IFT22 homolog, Rabl5 orthlog) | YH461(*tm1724*) | Insertion/Coding | + | - | - |
| *k04f10.2* (putative IFT-25 homolog) | TM1830(*tm1830*) | Deletion/Coding | + | - | - |
| *osm-1* (IFT172 homolog, WD repeat, IFT) | PR808(*p808*) | Unknown | - | + | + |
| *osm-1* (IFT172 homolog, WD repeat, IFT) | PR816(*p816*) | Unknown Deletion | - | + | + |
| *osm-5* (IFT88 homolog, polaris, IFT) | PR813(*p813*) | Substitution/Nonsense | - | + | + |
| *osm-6* (IFT52 homolog, IFT) | PR811(*p811*) | Splice site substitution | - | + | + |
| *rab-28* (RAB family, IFT27 homolog) | RB2484(ok3424) | Deletion/Coding | - | + | + |
|  | | | | | |
| **Bardet-Biedl Syndrome Complex Mutants** | | | | | |
| *bbs-1* (BBS1 ortholog, IFT) | VC837(*ok1111*) | Deletion/Coding | - | + | + |
| *bbs-2* (BBS2 ortholog, IFT) | VC1569(*ok2053*) | Deletion/Coding | - | + | + |
| *bbs-3* (BBS3 ortholog, IFT) | RB2509(*ok3472*) | Deletion/Coding | + | - | - |
| *bbs-4* BBS4 ortholog, IFT) | TM3038(*tm3038*) | Deletion/Coding | + | - | - |
| *bbs-5* (BBS5 ortholog, IFT) | VC1316(*gk537*) | Deletion/Coding | + | - | - |
| *bbs-8* (BBS8 ortholog, TPR protein, IFT) | MX52(*nx77*) | Deletion/Coding | - | + | + |
| *bbs-9* (BBS9 ortholog, IFT) | VC1062(*gk471*) | Deletion/Coding | - | + | + |
| *dct-14* (BBIP1 ortholog) | VC3240(*gk3188*) | Deletion/Coding | + | - | - |
| *k07c11.10* (BBS10 ortholog) | TM3304(*tm3304*) | Deletion/Coding | + | + | + |
| *osm-12* (bbs7, IFT) | MT3645(*n1606*) | Substitution/Nonsense | - | + | + |
|  | | | | | |
| **IFT Cargo Mutants** | | | | | |
| *ckr-2* (CCKBR ortholog) | LSC32(*tm3082*) | Deletion/Coding | + | - | - |
| *daf-25* (ANKMY2 ortholog, MYND domain) | DR2386(*m362*) | Deletion/Coding | + | - | - |
| *gpa-4* (G protein subunit alpha i3) | NL790(*pk381*) | Deletion/Coding | + | - | - |
| *gpa-15* (G protein alpha subunit) | NL797(*pk477*) | Deletion/Coding | + | - | - |
| *npr-16* (OPRK1 ortholog) | RB1365(*ok1541*) | Deletion/Coding | + | - | - |
| *npr-24* (SSTR5 ortholog) | VC2421(*ok3192*) | Deletion/Coding | + | - | - |
| *npr-32* (CCR2 ortholog) | RB1938(*ok2541*) | Deletion/Coding | + | - | - |
| *ocr-2* (TRPV5/6 family) | CX4544(*ak47*) | Deletion/Coding | + | - | - |
| *odr-3* (G protein alpha subunit) | MT4810(*n2046*) | Unknown | + | - | - |
| *odr-10* (GPCR) | CX3410(*ky225*) | Unknown | + | - | - |
| *osm-9* (TRPV5/6 family) | CX10(*ky10*) | Substitution/Nonsense | + | + | + |
| *osm-9* (TRPV5/6 family) | JY190(*yz6*) | Substitution/Nonsense | + | - | - |
| *osm-9* (TRPV5/6 family) | VC1262(*ok1677*) | Deletion/Coding | + | - | - |
| *pkd-2* (polycystin 2) | PT8(*sy606*) | Unknown | + | - | - |
| *tax-4* (CNGA2/3 ortholog) | VC3113(*ok3771*) | Deletion/Coding | + | - | - |
| *tub-1* (TUB like ortholog) | RB1600(*ok1972*) | Deletion/Coding | + | - | - |
|  | | | | | |
| **OSM-9 Interacting/Associated Proteins** | | | | | |
| *adp-1* (uncloned) | CX20(*ky20*) | Unknown | + | - | - |
| *akt-1* (serine/threonine kinase) | RB759(*ok525*) | Deletion/Coding | + | - | - |
| *let-60* (HRAS ortholog, GTPase) | SD551(*ga89*) | Substitution/Missense | + | - | - |
| *npr-1* (NPY1R ortholog) | CX4148(*ky13*) | Substitution/Nonsense | + | - | + |
| *npr-1* (NPY1R ortholog) | RB1330(*ok1447*) | Insertion/Coding | + | - | - |
| *ocr-1* (TRPV5/6 family) | CX4533(*ok132*) | Deletion/Coding | + | - | - |
| *ocr-3* (TRPV5/6 family) | RB1374(*ok1559*) | Unknown | + | - | - |
| *ocr-4* (TRPV5/6 family) | LX950(*vs137*) | Insertion/Coding | + | - | - |
| *odr-4* (ODR4 ortholog) | MT5300(*n2144*) | Unknown | + | - | - |
| *tax-2* (CNGB3 ortholog) | RB2464(*ok3403*) | Unknown | + | - | - |
| *tax-6* (PPPC3 ortholog) | RB1667(*ok2065*) | Insertion/Coding | + | - | - |
| *tph-1* (tryptophan hydrolase 1 ortholog) | MT14984(*n4622*) | Deletion/Coding | + | - | - |
| *trpa-1* (TRPA1 ortholog) | RB1052(*ok999*) | Deletion/Coding | + | - | - |
|  | | | | | |
| **Other Cilia Membrane Protein Mutants** | | | | | |
| *ccdc-149* (CCDC149 ortholog, coiled-coil protein) | VC1066(*gk456*) | Deletion/Coding | + | - | - |
| *cil-7* (myristoylated coiled-coil protein, leucine zipper domain) | TM5848(*tm5848*) | Deletion/Coding | + | - | - |
| *sto-1* (STOM ortholog) | TM1503(*tm1503*) | Deletion/Coding | + | - | - |
| *sto-2* (STOM ortholog) | TM1475(*tm1475*) | Deletion/Coding | + | - | - |
|  | | | | | |
| **Other Sensory Mutants** | | | | | |
| *che-7* (innexin homolog) | RB1834(*ok2373*) | Deletion/Coding | + | - | - |
| *gcy-35* (GUCY1B1 ortholog) | RB906(*ok769*) | Deletion/Coding | + | - | - |
| *inx-19* (innexin homolog) | CX6161(*ky634*) | Substitution/Missense | - | + | + |
| *osm-7* (Notch binding) | HA1857(*tm2256*) | Insertion/Coding | + | - | - |
| *osm-8* (mucin like protein) | RB2555(*ok3560*) | Deletion/Coding | + | - | - |
| *osm-10* (major sperm protein domain) | MT3641(*n1602*) | Unknown | + | - | - |
| *osm-13* (uncloned) | MT3664(*n1600*) | Unknown | + | - | - |
| *mec-4* (SCNN1B ortholog) | TU253(*u253*) | Deletion/Coding | + | - | - |
| *unc-7* (innexin homolog) | CB5(*e5*) | Substitution/Nonsense | + | + | + |
| *unc-9* (innexin homolog) | CB101(*e101*) | Splice site substitution | + | + | + |
|  | | | | | |
| **EMS Generated Mutants** | | | | | |
| TP236 (10nM Ivermectin resistant) | TP236(*ka30*) | Substitution/Nonsense | - | + | + |
| TP241 (50nM Abamectin resistant) | TP241(*ka35*) | Deletion/Coding | - | + | + |
| TP272 (10nM Ivermectin resistant) | TP272(*ka64*) | Substitution/Nonsense | - | + | + |
| TP274 (10nM Ivermectin resistant) | TP274(*ka66*) | Substitution/Missense | - | + | + |
| TP375 (10nM Moxidectin resistant) | TP375(*ka200*) | Substitution/Nonsense | - | + | + |
| TP378 (10nM Moxidectin resistant) | TP378(*ka201*) | Deletion/Frameshift | - | + | + |
| TP384 (10nM Moxidectin resistant) | TP384(*ka202*) | Substitution/Nonsense | - | + | + |
| TP386 (10nM Moxidectin resistant) | TP386(*ka203*) | Splice site substitution | - | + | + |
| TP388 (10nM Moxidectin resistant) | TP388(*ka204*) | Substitution/Nonsense | + | + | + |
|  | | | | | |
| **Miscellaneous Mutants** | | | | | |
| *c11e4.8* (uncloned) | TM11304(*tm11304*) | Deletion/Coding | + | - | - |
| *f41e7.2* (SLC9B1/2 ortholog) | RB2002(*ok2647*) | Deletion/Coding | + | - | - |
| *f59f5.7* (KNK ortholog, DOMON domain protein) | TM7257(*tm7257*) | Deletion/Coding | + | - | - |
| *ncs-4* (KCNIP2 ortholog) | TM4409(*tm4409*) | Deletion/Coding | + | - | - |
| *nep-15* (neprilysin metallopeptidase family) | VC2361(*ok3079*) | Deletion/Coding | + | - | - |
| *nmur-1* (NMUR1 ortholog) | RB1288(*ok1387*) | Deletion/Coding | + | - | - |
| *trk-1* (neurotrophin receptor) | TM10162(*tm10162*) | Deletion/Coding | + | - | - |
